# Impacts of climate variability and adaptation strategies on crop yields and soil organic carbon in the US Midwest

**DOI:** 10.1371/journal.pone.0225433

**Published:** 2020-01-28

**Authors:** Lin Liu, Bruno Basso

**Affiliations:** 1 Department of Earth and Environmental Sciences, Michigan State University, East Lansing, Michigan, United States of America; 2 W.K. Kellogg Biological Station, Michigan State University, Hickory Corners, East Lansing, Michigan, United States of America; Beijing Normal University, CHINA

## Abstract

Climate change is likely to increase the frequency of drought and more extreme precipitation events. The objectives of this study were i) to assess the impact of extended drought followed by heavy precipitation events on yield and soil organic carbon (SOC) under historical and future climate, and ii) to evaluate the effectiveness of climate adaptation strategies (no-tillage and new cultivars) in mitigating impacts of increased frequencies of extreme events and warming. We used the validated SALUS crop model to simulate long-term maize and wheat yield and SOC changes of maize-soybean-wheat rotation cropping systems in the northern Midwest USA under conventional tillage and no-till for three climate change scenarios (one historical and two projected climates under the Representative Concentration Path (RCP) 4.5 and RCP6) and two precipitation changes (extreme precipitation occurring early or late season). Extended drought events caused additional yield reduction when they occurred later in the season (10–22% for maize and 5–13% for wheat) rather than in early season (5–17% for maize and 2–18% for wheat). We found maize grain yield declined under the projected climates, whereas wheat grain yield increased. No-tillage is able to reduce yield loss compared to conventional tillage and increased SOC levels (1.4–2.0 t/ha under the three climates), but could not reverse the adverse impact of climate change, unless early and new improved maize cultivars are introduced to increase yield and SOC under climate change. This study demonstrated the need to consider extreme weather events, particularly drought and extreme precipitation events, in climate impact assessment on crop yield and adaptation through no-tillage and new genetics reduces yield losses.

## 1. Introduction

Climate change entails changes in climate variability and in the frequency of extreme weather events. To curb the impact of climate variability and change on crop yield, adaptation strategies need to be developed and evaluated. US Midwest has experienced increases in daily average temperature, increased precipitation in mid spring and early summer, and decreased precipitation in mid-to-late summer and early fall since the 1980s [1, 2]. It is projected that the Midwest will continue to experience increased temperature, elevated CO_2_ concentration and variable precipitation with more frequent drought and storm events [3]. The changing climate, particularly more frequent occurrence of extreme weather events, have caused declines in yield of summer grain crop at the national level [4, 5].

No-till has been studied and promoted to sustain crop production in the Midwest in wake of the changing climate [6, 7]. Climate change, particularly the warming temperature and varying precipitation, has a negative effect on grain yield and soil organic carbon (SOC). The increased temperature under climate change leads to faster crop development which reflects in yield declines and quicker soil carbon mineralization [8, 9]. The unpredictable precipitation frequency imposes water deficiency stress on crop growth, which in turn reduces biomass accumulation and residue return to soils [9]. No-till practice may reduce such adverse climate impact on yield and SOC by conserving soil structure, storing carbon and nutrient and enhancing water retention [10, 11, 12]. Field experiments in the Midwest showed that no-till fields were able to produce higher grain yield and to store more carbon compared to the conventionally tilled fields [13, 14, 15].

The interconnections among grain yield, SOC, tillage management and climate change, however, have been overlooked. SOC dynamics is determined by mineralization rates, which is affected by temperature, soil moisture, and return of residue, which is affected by climate and tillage management [9, 16, 17]. A few studies have focused on the feedback between climate and SOC [18, 19, 9] provided the first study in the literature where the concomitant effects of yield decline and SOC losses under climate change were addressed in a systems feedback loop.

Climate change and the increase of extreme weather events will undoubtedly have an adverse impact on agricultural production in the US Midwest unless new adaptation strategies are implemented. Adaptation strategies, such as planting the existing cultivar earlier and planting improved cultivars, are likely to be considered by growers [20]. Early planting can take advantage of the warming temperature in the beginning of the growing season and avoid late-season heat stress [21, 22]. Improved cultivars with new traits, longer duration and more efficient kernel set, have higher yield potential and may mitigate the climate impact [23, 24]. Yet, the effectiveness of the adaptation strategies on yield improvement and SOC conservation has not been evaluated systematically considering extreme events under climate change and tillage practices.

Crop simulation models consider the interactions between the four major components of a cropping system (i.e. climate, genotypes, management, and soil). Models can be used to understand the impact of climate change and extreme weather on cropping systems, including grain yield and soil carbon dynamics, and to assess the effectiveness of climate adaptation strategies [25, 26]. The Systems Approach to Land Use Sustainability (SALUS) model [27] has been applied to assess the response of cereal and non-cereal crop production across the globe under different climate scenarios [28, 29, 30]. Few studies, however, have explicitly focused on simulating extreme weather events under future climate change to access the impact of those events on crop yield and SOC change. Climate variability and extreme weather events could be reflected in the projected climate scenarios through forcing a higher standard deviation value of climatic variables, or through changing the duration of dry and wet spells [31, 32, 33].

The overarching goal of this study was to evaluate adaptation strategies to mitigate the effects of extended drought and heavy precipitation on yields and SOC in a US Midwest maize-soybean-wheat (*Zea mays-Glycine max-Triticum aestivum*) rotation under current and future climate projections. More specifically, we aimed to (i) assess the effect of tillage on grain yield and SOC under extended drought followed by a heavy rainfall event in two different phenological stages (when the maize has 6 fully extended leaves (V6), and during the reproductive stage, when maize is reaching tassel emission (VT) later in the season) for historical climate, (ii) evaluate the effects of tillage systems on yield and SOC dynamics under the projected climate with drought and extreme precipitation events, and (iii) evaluate the effectiveness of two climate adaptation measures (e.g. anticipating planting date of existing cultivars and improved cultivars), on mitigating the adverse impact of extreme weather events.

## 2. Materials and methods

### 2.1 Description of the study sites

**Fig 1:**
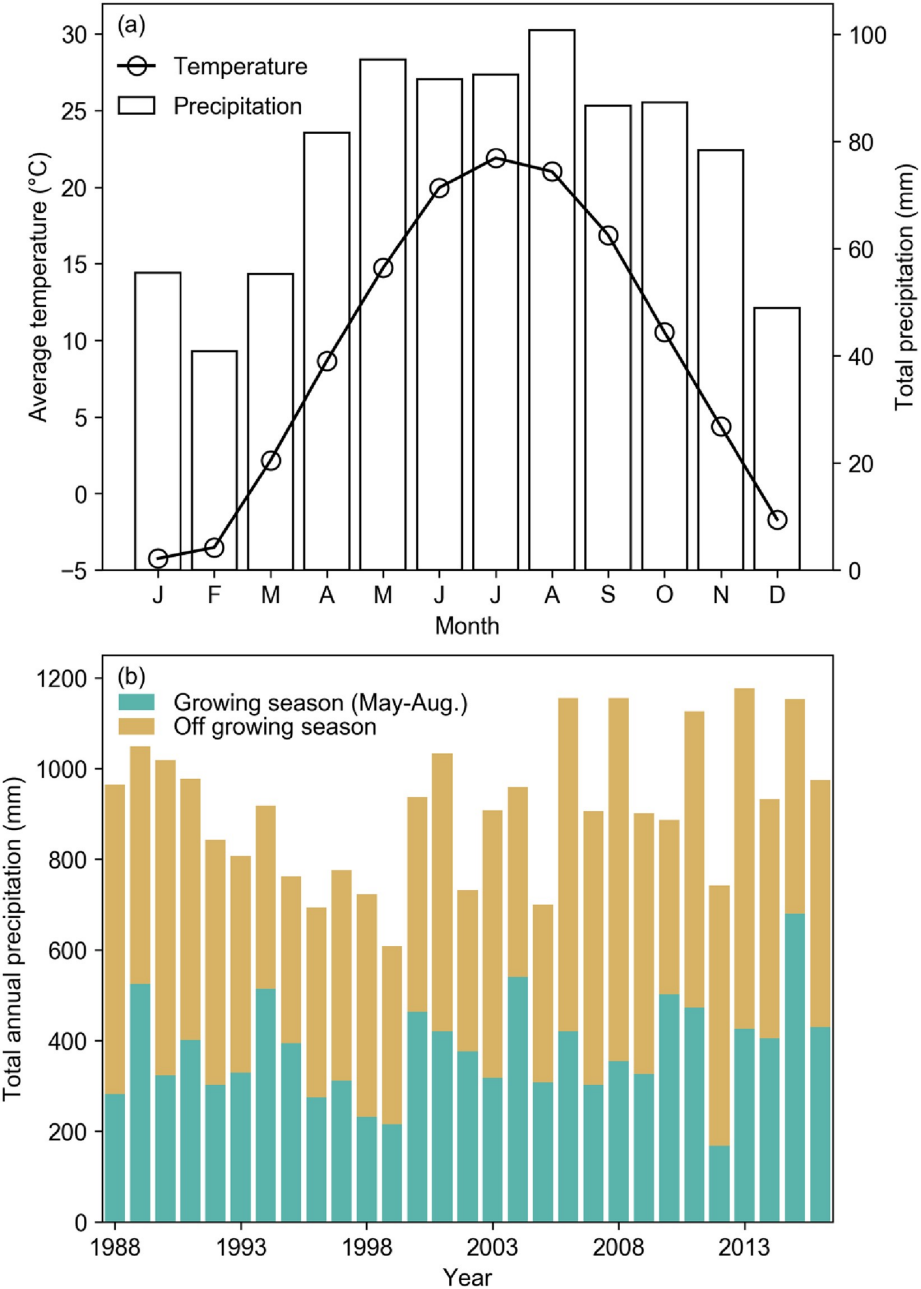
(a) Monthly distribution of average temperature and total precipitation, and (b) annual total precipitation in growing seasons (May-August) and off growing season in 1988–2016.

The maize-soybean-winter wheat rotation system is part of the field experiment conducted at the W.K. Kellogg Biological Station (KBS) Long Term Ecological Research (LTER) site in southwestern Michigan, United States (85°24’W, 42°24’N, 288 m a.s.l.). The soils of the experimental site are Kalamazoo (fine-loamy) and Oshtemo (coarse-loamy) series [34]. The climate of the study site is temperate with cold and wet winters and warm and wet summers (Fig 1a). The average temperature between 1988 and 2016 was 9.3 °C (Fig 1a). The average annual total precipitation was 915 mm with large inter-annual variations (standard deviation of the annual total precipitation was 154 mm; Fig 1b).

KBS-LTER was established in 1988 to study the ecology of intensively managed field crops and ecological processes occurring in the surrounding landscape [35]. The experiments use a randomized complete block design with six replications. The field experiments include annual cropping systems under four treatments with varying management intensity, perennial cropping systems and unmanaged ecosystems. Each of the experiments has six 1-ha replicates [35]. Two weather stations are present at the experimental site. In this study, we analyzed the annual maize-soybean-wheat rotation systems under conventional tillage and no-till treatments. The conventional treatment represents typical farming practices in the region with conventional tillage (chisel plow within 10 days before planting), fertilization and herbicide applications. The average nitrogen fertilizer application rate in 1989–2016 was 156 kg/ha for maize and 86 kg/ha for wheat. Soybeans are not fertilized. The no-till treatment has the same level of chemical input, but it is under No-till since 1989. For both treatments, maize is seeded in early-to-mid May, soybean is seeded in late May to early June, and wheat is seeded in fall after soybean are harvested. Maize and soybean are harvested in the early fall and wheat is harvested in July.

### 2.2 Overview of the crop simulation model and model validation

#### 2.2.1 Overview of the SALUS model

In this study, we used the System Approach for Land Use Sustainability (SALUS) model, a process-based crop model designed to simulate the crop-soil-climate-management interactions at a daily time-step intervals [36, 27, 37]. SALUS derives from the CERES model [38] with major differences in the algorithms simulating the carbon, nitrogen and phosphorus cycles, water balance, tillage, kernel number and weight determination. Unlike the CERES model simulation of a single crop cycle, the SALUS model simulates rotational cropping systems continuously for multiple years. The SALUS model uses daily weather (minimum and maximum temperature, precipitation, and solar radiation), soil layer information (e.g. layer depth, drained upper limit, lower limit, bulk density and organic carbon), management information (e.g. planting date, fertilizer type and rate, and harvesting date), and genotype characteristics to simulate crop growth and development, nutrient cycle and water balance. The model consists of three interconnected modules: the crop module, the nutrient cycle module and the water balance module [27, 36].

SALUS has two types of crop models, a simple model and a complex model. A simple model is based on a generic crop specific curve describing leaf area index (LAI) development in relation to thermal time to simulate crop developmental phases and potential biomass accumulation. The complex crop model is similar to CERES and uses genetic coefficients. Biomass accumulation is related to crop radiation use efficiency, leaf area development, with growth reduction in presence of nutrient and water stress. The processes of the SOC dynamics include residues and root decomposition, soil mineralization, immobilization and conversion to gaseous forms. The SOC pool sizes initialization procedure is documented in [39]. The water balance module uses Ritchie’s method to simulate infiltration, drainage, evapotranspiration, runoff and water redistribution [40, 41, 42].

SALUS is designed to simulate tillage systems and their impact on bulk density, hydraulic conductivity, infiltration and SOC. Different tillage systems can be simulated to account the effects of tillage depth and tillage intensity and soil inversion effects on water and nutrient fluxes. A detailed description of the tillage models is available in [36].

#### 2.2.2 SALUS model validation

The SALUS model has been validated for grain yield, evapotranspiration, drainage and SOC in the cropping systems under various treatments at KBS [27]. In this study, we validated the model by comparing the simulated yield against the observed grain yield in the maize-soybean-wheat fields under conventional and no-till treatments from 1989–2016 at KBS. Observed annual grain yield and the input for the model, including soil, weather and management, were obtained from the KBS LTER website (https://lter.kbs.msu.edu/datatables). We also compared the simulated SOC change in 1989–2006 under the two treatments to the reported change at the KBS LTER site [43]. The model accuracy was evaluated using linear regression with simulated yield as the independent variable and observed yield as the dependent variable. Additionally, we used the root mean square of deviation (RMSD) and mean absolute percentage error (MAPE) between the simulation and the observation to evaluate model accuracy.

### 2.3 Simulation experiments

**Fig 2:**
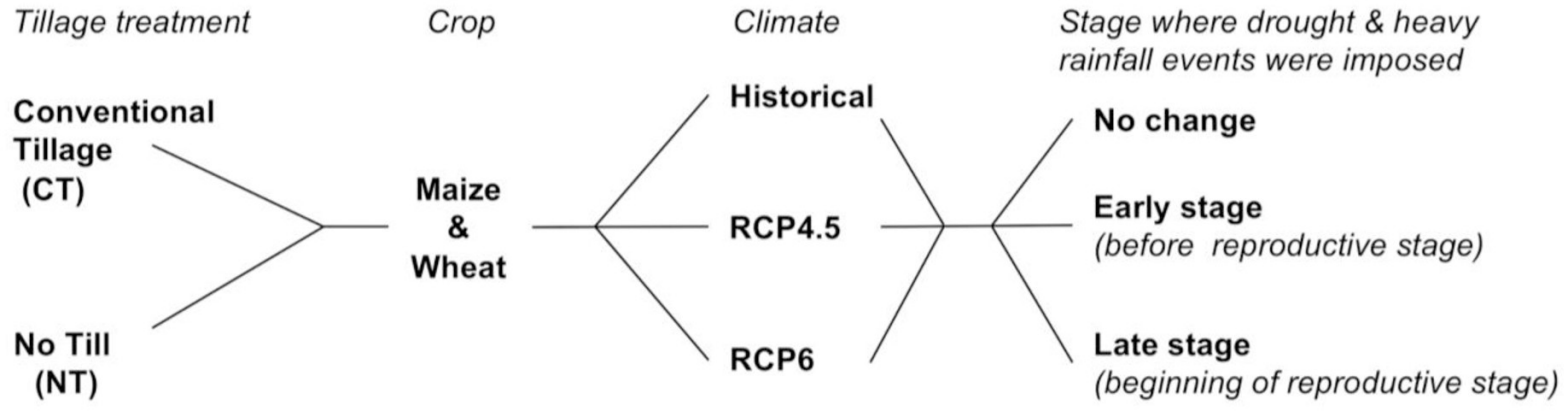
Illustration of the simulation experiments studying the impact of tillage treatments and climate change, considering climate scenarios and precipitation variability at crops’ early and late stages, on the yields of maize and wheat and SOC of the maize-soybean-wheat rotational cropping system.

We used the validated SALUS model to simulate the impact of tillage treatments and climate change on the yields and top 15cm SOC of maize-soybean-wheat rotation cropping systems. The rotational systems consisted of maize-soybean rotations in the first four years and maize-soybean-wheat rotations for 24 years. The tillage treatments in the simulation experiments were conventional tillage and no-till. The crops of interest in this paper were maize and wheat. For climate change, we considered changes in both the mean and the variability in the simulations (Fig 2).

We included three climate scenarios: the historical and the two medium stabilization representative concentration paths (RCPs). The historical climate was the observed weather data in 1989–2016. The two RCPs were RCP4.5 and RCP6. The radiative forcing was projected to stabilize at 4.5 W m^-2^ after the end of the 21^st^ century for the RCP4.5, whereas it would be stabilized at 6 W m^-2^ for the RCP6 scenario [44]. In this study, CO_2_ was set at 525 ppm and temperature (both maximum and minimum temperature) were raised by 3 °C under the projected RCP4.5 climate. Precipitation was increased by 10% in winter and spring, while it was decreased by 10% in the summer under the RCP4.5 climate [45, 46]. Under the projected RCP6 climate, CO_2_ was raised to 625 ppm during the simulated years, temperature was increased by 4 °C, and precipitation was increased by 15% in winter and spring, while it was decreased by 15% in the summer [45, 46] (Table 1).

**Table 1:** Descriptions of the climate scenarios in the study.

Climate scenarios	Descriptions					
	CO_2_ (ppm)	Temperature (°C)	Changes in seasonal precipitation amount			Precipitation frequency scenarios^†^
			*Spring*	*Summer*	*Winter*	
**Historical (baseline)**	375	N/A^+^	N/A	N/A	N/A	N/A
Historical climate with *early* drought	375	N/A	N/A	N/A	N/A	Early growth stage
Historical climate with *late* drought	375	N/A	N/A	N/A	N/A	Late growth stage
**RCP4.5**	525	+3	10% more	10% less	10% more	N/A
RCP4.5 climate with *early* drought	525	+3	10% more	10% less	10% more	Early growth stage
RCP4.5 climate with *late* drought	525	+3	10% more	10% less	10% more	Late growth stage
**RCP6**	625	+4	15% more	15% less	15% more	N/A
RCP6 climate with *early* drought	625	+4	15% more	15% less	15% more	Early growth stage
RCP6 climate with *late* drought	625	+4	15% more	15% less	15% more	Late growth stage

^†^Precipitation distribution was manipulated during wheat and maize early (vegetative stage before the reproductive stage) or late (beginning of the reproductive stage) development stages

^+^N/A means that the climatic variable was not changed compared to the historical climate

We explicitly considered precipitation variability in the simulations. For the historical and the two projected climate scenarios, we prepared three precipitation frequency manipulation scenarios to represent drought and extreme rainfall under climate change. The three precipitation scenarios were i) no changes in rainfall variability, and prolonged drought period followed by a heavy rainfall event during crops’ critical stages—vegetative stage in the (ii) early part of season *versus* (iii) reproductive stage in the late part of season. In the first precipitation scenario, the frequency of daily precipitation remained unchanged compared to the historical climate. In the other two precipitation scenarios, we imposed a drought period during the critical stages by eliminating daily rainfall such that the number of consecutive dry days (receiving less than 1 mm precipitation) in the early and the late seasons to be the maximum number of consecutive dry days in the respective seasons in 1988–2017. The extreme rainfall event in the scenario was created by moving the cumulative daily rainfall in the drought period to the day after the imposed drought ended. The maximum number of consecutive dry days for wheat, both early season and late season, was 20 days. The maximum number of consecutive dry days for maize early season was 23 days and 20 days for late season (S1 Fig). In the study site, the critical early season is March for wheat (Feekes 7–9) and mid-June to mid-July for maize (V5-V10). The critical late season is June for wheat (Feekes 10.1–10.5) and mid-July to mid-August for maize (vegetative-tasseling to early grain-filling stage).

We first ran the validated SALUS model with the reported crop types and current management practices under 18 scenarios combining three climate scenarios, three precipitation frequency scenarios, and two tillage treatments. Then, we tested two climate adaptation practices by running the SALUS model for two RCP climates, the three precipitation frequency scenarios and the two tillage treatments (Fig 2 and Table 1). The two climate adaptation practices were i) planting maize 15 days earlier than the current planting dates and ii) planting early a maize cultivar that has a 250 degree-day longer duration, more kernels per ear (750 vs 600 current), and increased kernel set efficiency (about 20% more efficient). We followed [23] to determine the two adaptation strategies and to parameterize the improved maize cultivar in the SALUS model. We used the simple approach of the model to simulate soybean and the complex approach to simulate maize and wheat.

### 2.4 Statistical analysis

We report the response of grain yield of maize and wheat to climate change, treatments and adaptation practices in the simulated 24 years (i.e. eight maize-soybean-wheat rotational cycles) and SOC change in the simulated 28 years.

To test the overall effect of tillage, climate and their interactions on grain yield, we performed a two-way analysis of variance (ANOVA) with tillage and climate as fixed factors and replicate and year as random factors. In the two-way ANOVA, tillage consisted of no-till and conventional tillage treatments. Climate consisted of nine treatments combining three greenhouse gas emission paths (historical, RCP4.5 and RCP6) and three precipitation frequency scenarios (precipitation frequency was not altered, and was altered at early versus late growth stage). To determine the significant difference in yield and SOC between the tillage treatments, we performed one-way ANOVA followed by a t-test when tillage factor was significant at P = 0.05. Similarly, we performed one-way ANOVA followed by Tukey’s honestly significant difference as a post hoc test to assess the significant difference in yield and SOC between the precipitation frequency scenarios, when the tested factor was significant at P = 0.05. We used the R package to perform the statistical test [47].

## 3. Results

### 3.1 SALUS model evaluation

**Fig 3:**
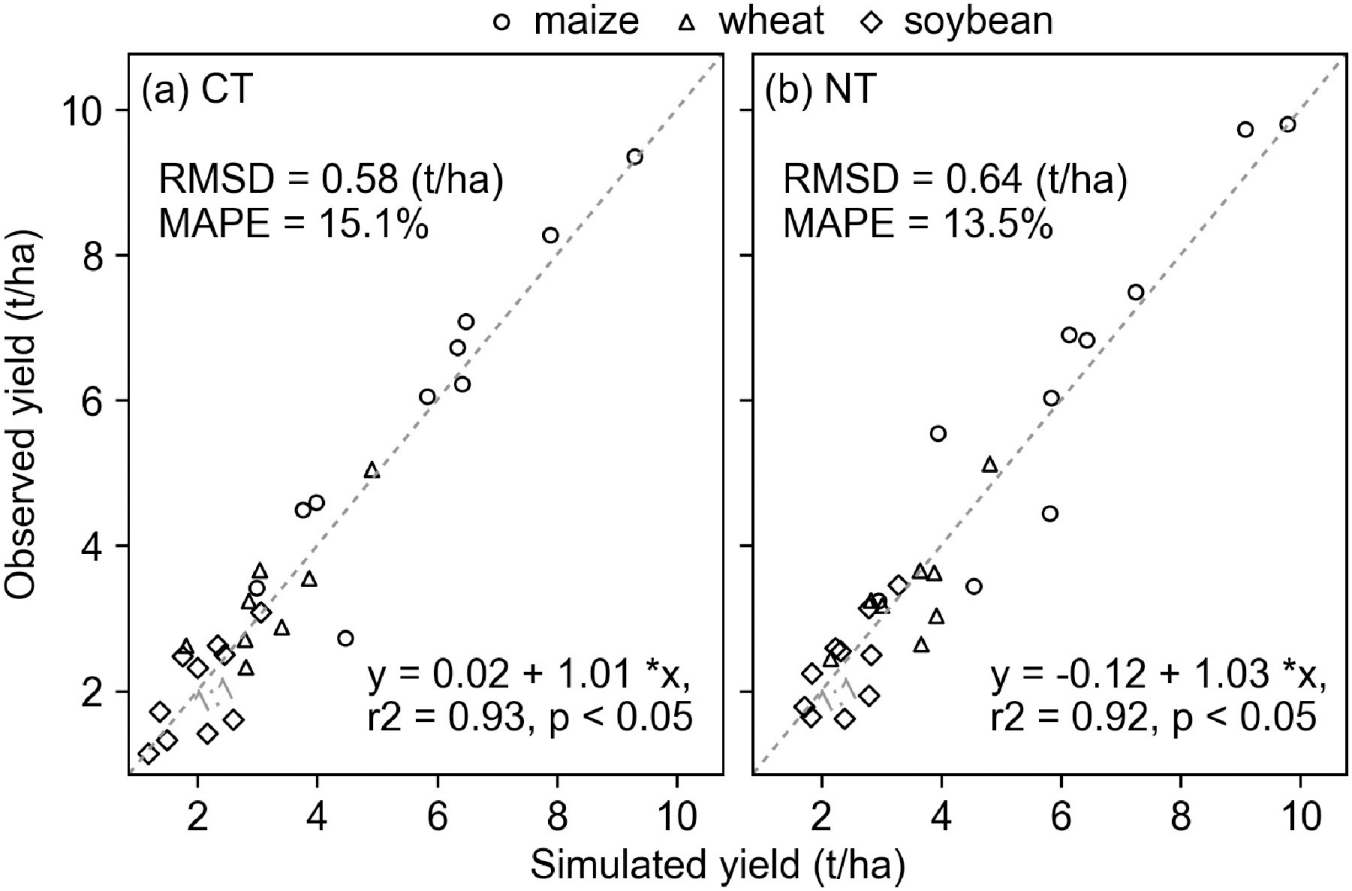
Comparisons between the simulated and the observed grain yield in the maize-soybean-wheat rotation system under (a) conventional (CT) and (b) no-till (NT) treatments at the Kellogg Biological Station in 1989–2016.

**Fig 4:**
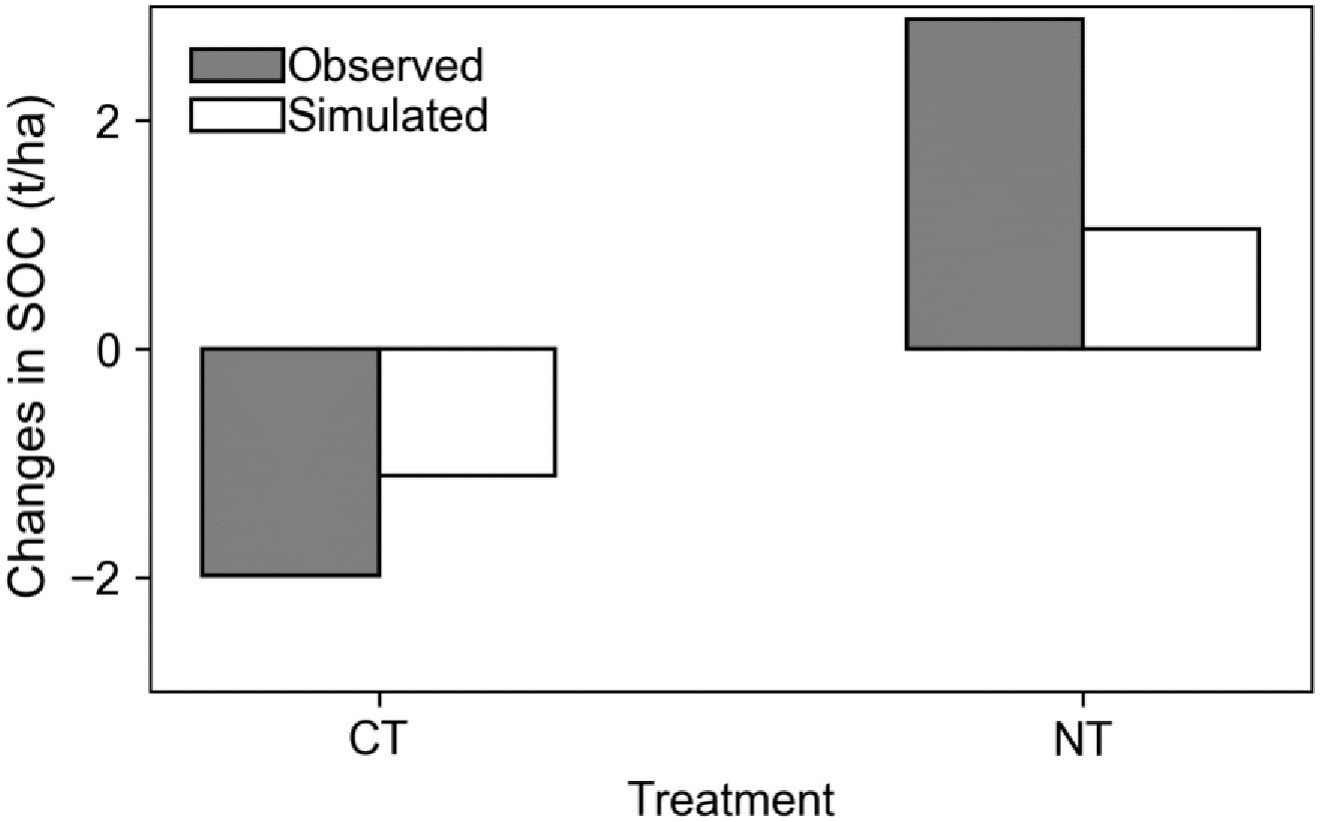
Comparisons between simulated SOC changes in 1989–2006 and observed change in 1988–2006 in the maize-soybean-wheat rotation system under conventional (CT) and no-till (NT) treatments at the Kellogg Biological Station (the observation was from [43]).

The SALUS model simulated the long-term yield of maize and wheat, and SOC under conventional and no-till treatments reasonably well. There was a significant correlation between the simulated and observed grain yield across the three crops for the conventional and no-till treatments. The root mean square of deviation (RMSD) value between simulated and observed yield under conventional tillage was 0.58 t/ha and was 0.64 t/ha for the no-till treatment. The mean absolute percentage error (MAPE) was 15.1% and 13.5% for the two treatments, respectively (Fig 3). The simulated changes in SOC in 1989–2006 under each of the treatments were comparable to the reported changes (Fig 4).

### 3.2 Maize grain yield response to tillage treatments and climate change

**Fig 5:**
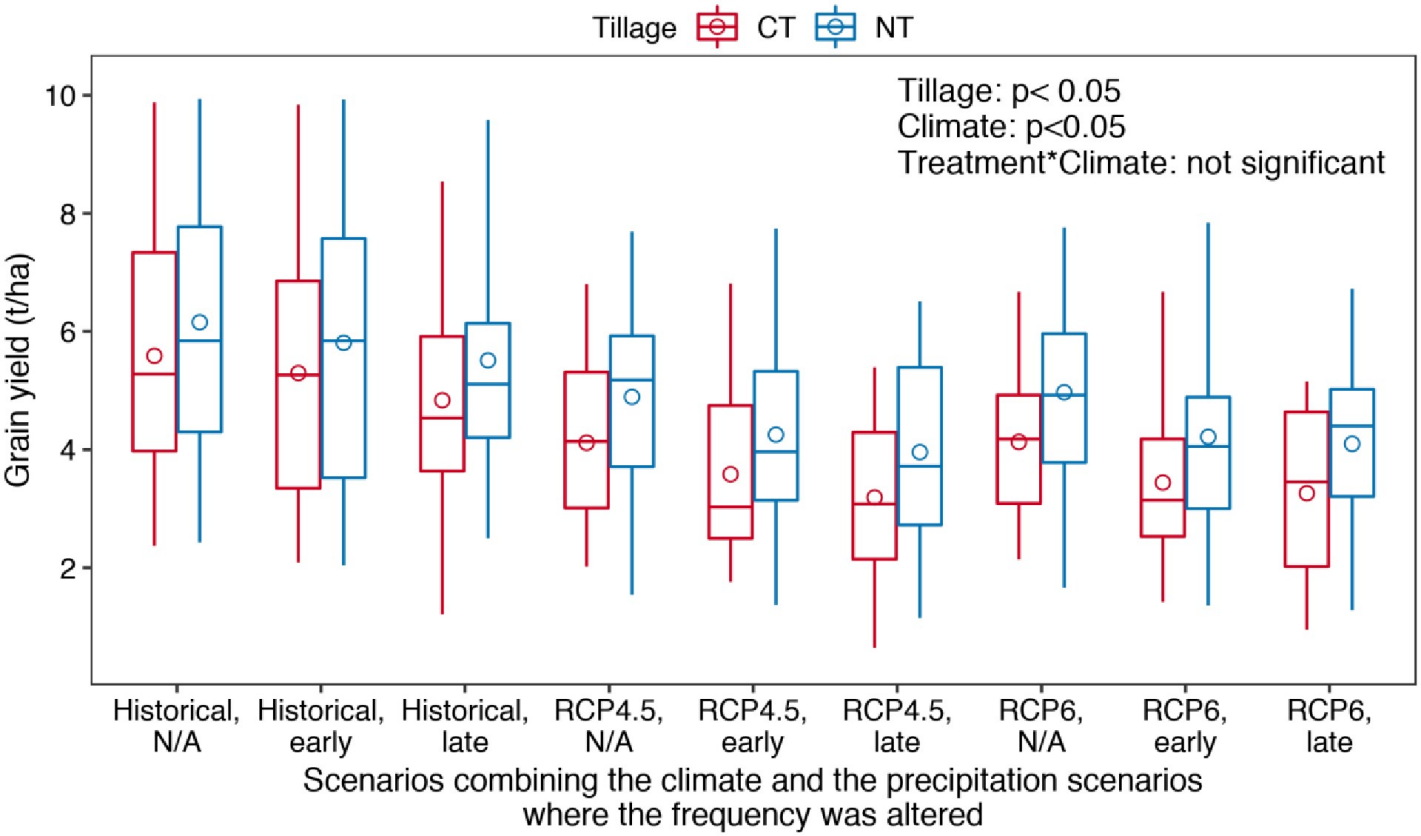
Distribution of the simulated maize grain yield under conventional (CT) and no-till (NT) treatments in the simulated 24 years under the nine climate treatments that combines three greenhouse gas concentration paths scenarios (historical, RCP4.5 and RCP6) and three precipitation frequency scenarios (no changes in precipitation frequency (N/A), and drought and heavy rainfall imposed for early or late growth stage) (p values are from the two-way ANOVA analysis; the top and the bottom of the box represent the first and the third quartile values, respectively; the line within the box presents the median value; the top and the bottom ends of the whiskers represent the maximum and the minimum values, respectively; the circle represents the mean value).

Both tillage treatments and climate treatments had a significant (p < 0.05) impact on maize grain yield but the interaction between the two factors was not significant (p > 0.05) (Fig 5).

Within each scenario that combined historical and the two projected climates and the three precipitation frequency scenarios, maize grain yield was higher under no-till treatment than conventional treatment. The yield benefit under no-till treatment was statistically significant (p < 0.05) for the six scenarios under RCP4.5 and RCP6 climates but insignificant (p > 0.05) for the historical climates, regardless of the imposed drought and heavy rainfall events (S2 Fig).

Under historical climate, the average maize yield in the simulated 24 years was 5.58 t/ha for conventional treatment and 6.15 t/ha for no-till treatment. With early-season drought and storm events, the average yield reduction was about 5% from the average yield under historical climate for the two tillage treatments. With late-season drought and storm events, the average yield reduction was 13% from the average yield under historical climate for conventional tillage and 10% for no-till treatment (Fig 5 and S4 Fig).

Under the RCP4.5 climate without changing precipitation frequency, maize yield declined to an average of 4.12 t/ha for conventional treatment, and to an average of 4.89 t/ha for no-till treatment (Fig 5). Early-season extreme precipitation events led to about 13% yield reduction for both treatments under RCP 4.5 climate. Late-season extreme events led to 22% and 19% reduction for conventional and no-till treatment, respectively, under the RCP 4.5 climate (Fig 5 and S4 Fig).

Under the RCP6 climate, the average yield in the simulated 24 years was 4.13 t/ha and 4.97 t/ha for conventional and no-till treatments, respectively (Fig 5). Early drought and storm events caused 17% yield loss for conventional treatment and 15% for no-till treatment when compared to the yield under RCP6 climate, whereas late drought and storm events caused 21% and 17% yield reduction from the yield under RCP6 climate, respectively (Fig 5 and S4 Fig).

### 3.3 Wheat grain yield response to tillage treatments and climate change

**Fig 6:**
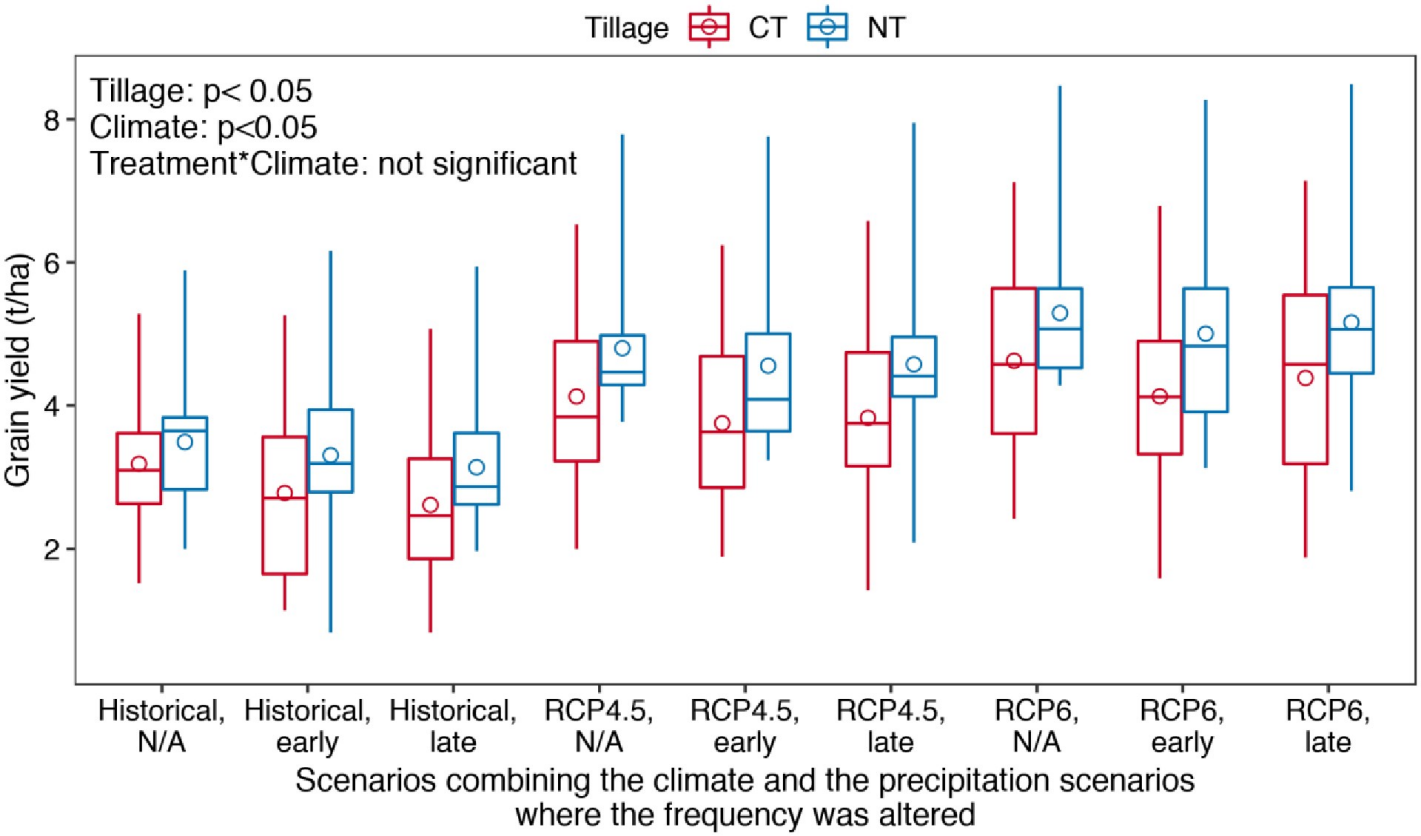
Distribution of the simulated wheat grain yield under conventional (CT) and no-till (NT) treatments in the simulated 24 years under the nine climate treatments that combines three greenhouse gas concentration paths scenarios (historical, RCP4.5 and RCP6) and three precipitation frequency scenarios (no changes in precipitation frequency (N/A), and drought and heavy rainfall imposed for early or late growth stage) (p values are from the two-way ANOVA analysis; the top and the bottom of the box represent the first and the third quartile values, respectively; the line within the box presents the median value; the top and the bottom ends of the whiskers represent the maximum and the minimum values, respectively; the circle represents the mean value).

The simulated wheat yield ranged from 0.83 to 8.49 t/ha over the 24 years across the two treatments and the nine climate scenarios. Wheat yield increased under the two projected RCP climates compared to the historical climate. Both tillage treatments and climate treatments had a significant (p < 0.05) impact on the grain yield but the interaction between the two factors was not significant (p > 0.05) (Fig 6).

Under the historical climate scenario, the simulated average wheat yield was 3.19 t/ha for conventional treatment and 3.49 t/ha for no-till treatment (Fig 6). For conventional treatment, the extreme precipitation events imposed at either early or late stage significantly reduced wheat grain yield, by about 13% and about 18%, respectively (p < 0.05) (S6a, S7a and S7b Figs). The reduction caused by early-season extreme precipitation events was insignificant for the no-till treatment (yield reduction was about 5%, p > 0.05) (S6d, S7c and S7d Figs) whereas the late-season extreme events caused significant wheat yield loss (by about 10%, p < 0.05) for the no-till treatment (S6d, S7c and S7d Figs).

For RCP4.5 climate, the simulated average yield was 4.13 t/ha and 4.80 t/ha under conventional and no-till treatments, respectively (Fig 6). With early-season extreme precipitation events, the yield was significantly reduced by 9% under conventional treatment and by about 5% under the no-till treatment (insignificant at P = 0.05). Late-season extreme events caused significant yield reduction (by 7%, p < 0.05) under conventional treatment but insignificant reduction (by less than 5%, p > 0.05) under no-till treatment (S6b, S6e and S7b–S7d Figs).

For RCP6 climate, the simulated average yield was 4.62 t/ha and 5.29 t/ha for conventional treatment and no-till treatment, respectively (Fig 6). Early-season extreme precipitation events led to significant yield reduction (by 11%) from the average yield under RCP6 climate under the conventional treatment (p < 0.05) and insignificant yield reduction (by about 5%) under the no-till treatment (p > 0.05). Similarly, late-season droughts and storm events led to significant yield reduction under the conventional treatment (by 5%, p < 0.05) but insignificant yield loss under the no-till treatment (by 2.5%, p > 0.05) (S6c, S6f and S7b–S7d Figs).

### 3.4 Response of SOC to tillage treatments and climate change

**Fig 7:**
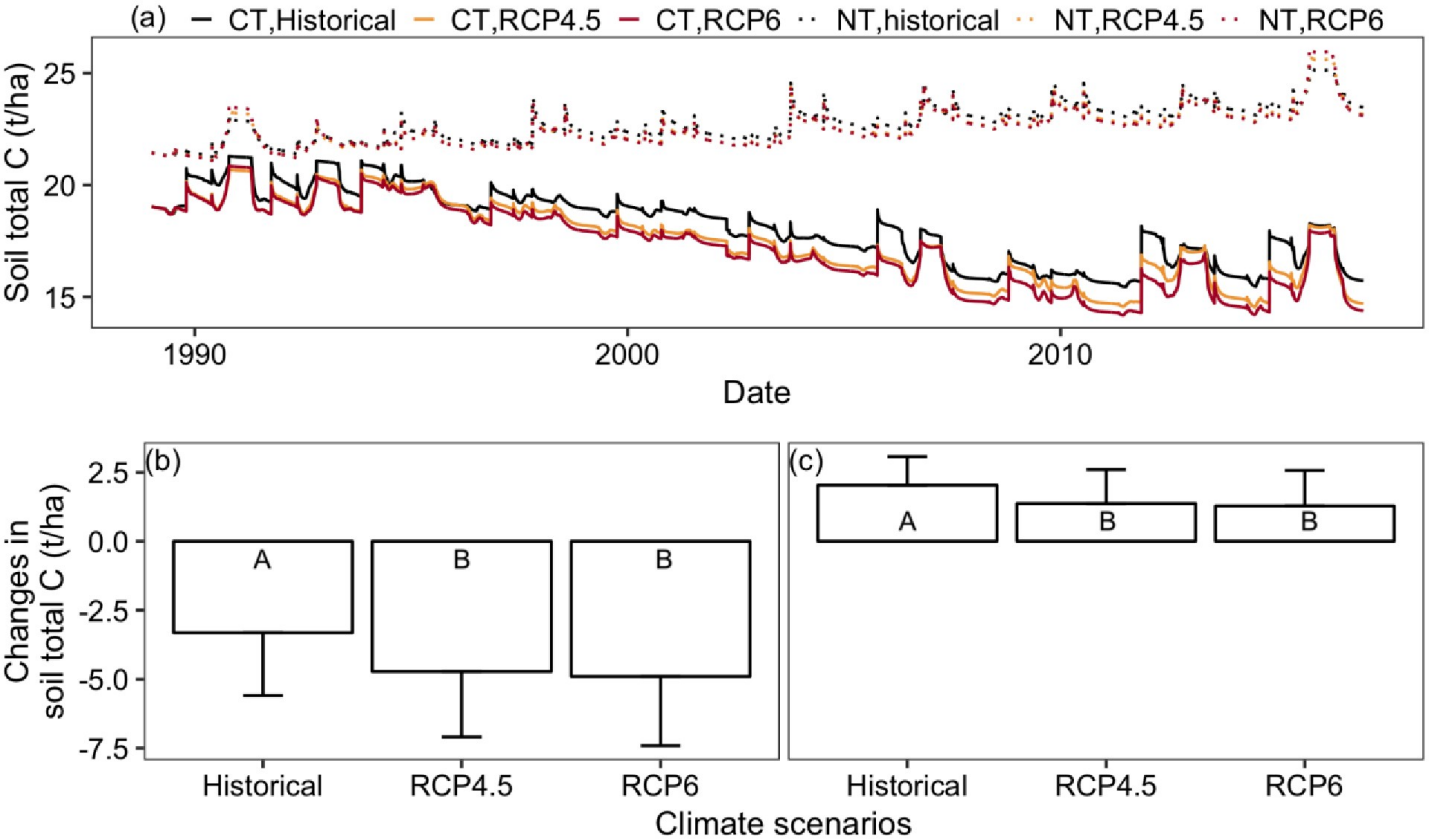
(a) Average SOC across the six replicates under the two treatments, conventional tillage (CT) and no-till (NT), during the simulated 28 years for historical, RCP4.5 and RCP6 climates; (b) Average and standard deviation of SOC loss in the simulated 28 years under conventional treatment; (c) Average and standard deviation of SOC gain in the simulated 28 years under no-till treatment (bars represent the standard deviation values; climate scenarios sharing the same letter are not significantly different at P = 0.05 using Tukey’s honestly significant difference test).

SOC for the top 15cm layer decreased in the simulated 28 years under conventional treatment while it increased under no-till treatment across the different climate scenarios. SOC decreased more under the projected climate than under the historical climates with conventional treatment, whereas SOC gain was less under the projected climates than under the historical climate with no-till treatment (Fig 7a). Across the six replicates in conventional treatment, the average SOC loss was 3.31 t/ha with standard deviation of 2.28 t/ha under the historical climate (Fig 7b). SOC loss was significantly greater under RCP4.5 and RCP6 climates (average loss was about 5 t/ha, p < 0.05), compared to the historical climate. Regarding the no-till treatment, SOC was increased to an average of 2.04 t/ha with standard deviation of 1.04 t/ha across the six replicates under the historical climate (Fig 7c). The SOC gain was significantly less under the projected climate (p <0.05), with an average increase of less than 1.4 t/ha under the projected climates (Fig 7c). For both treatments, the SOC change was insignificantly (p > 0.05) different between RCP4.5 and RCP6 climate (Fig 7b and 7c).

### 3.5 Maize yield and SOC under climate adaptation strategies

**Fig 8:**
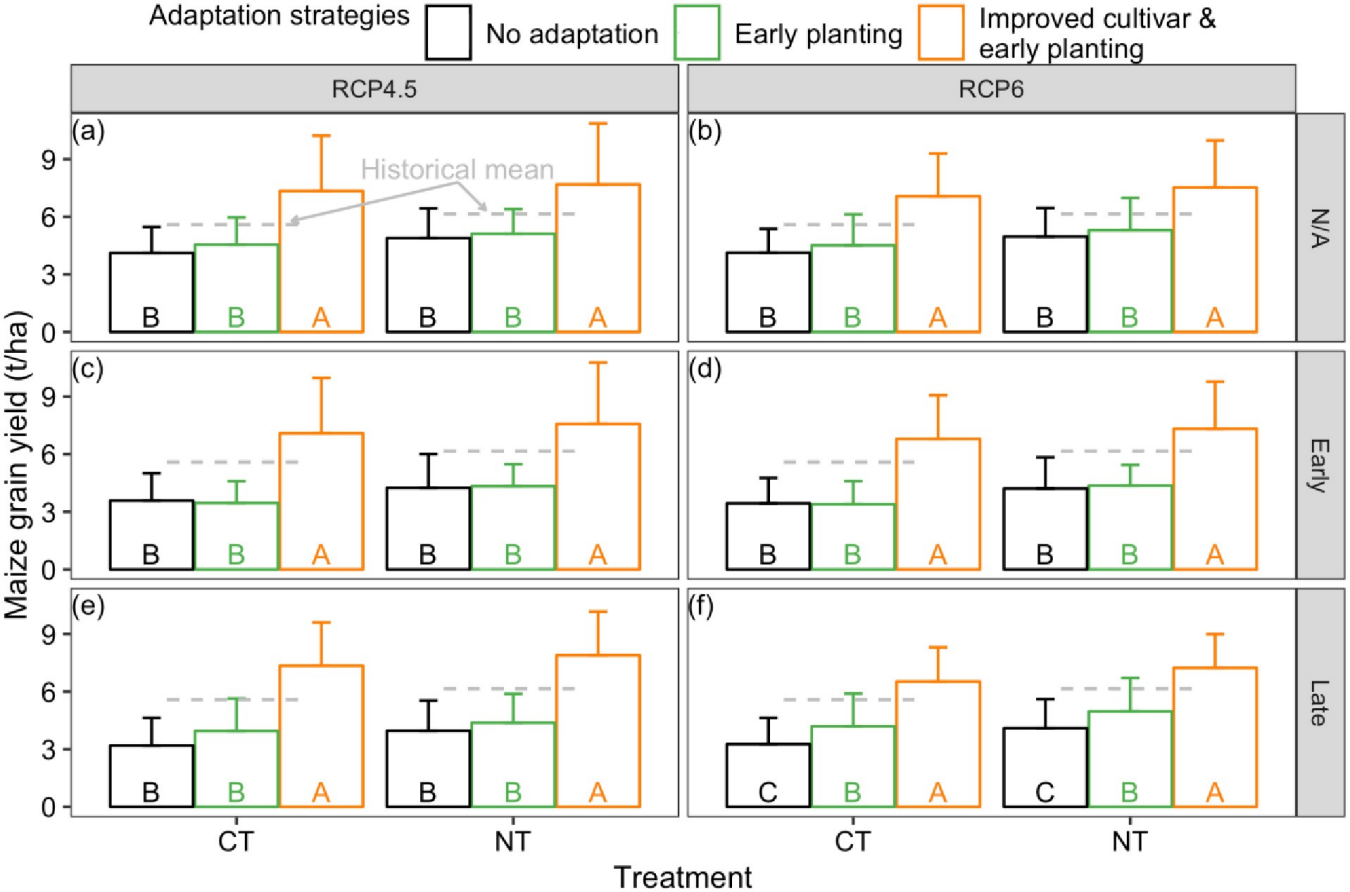
Average and standard deviation of the simulated maize yield with and without climate adaptation strategies under conventional tillage (CT) and no-till (NT) in the 24 years for various projected climate scenarios: (a-b) RCP4.5 and RCP6 climate without changing precipitation frequency, (c-d) RCP4.5 and RCP6 climate combined with changing precipitation frequency at maize and wheat early growth stage, (e-f) RCP4.5 and RCP6 climate combined with changing precipitation frequency at maize and wheat late growth stage (error bars represent the standard deviation; adaptation strategies sharing the same letter are not significantly different at P = 0.05 using Tukey’s honestly significant difference test within the tillage treatment).

Maize yield is slightly increased (by less than 12%) with the 15-day earlier planting practices compared to the no adaptation practices across the two tillage treatments and across the projected climate scenarios. Maize yield improvement by this adaptation measure was only significant (p <0.05) under RCP6 with extreme rainfall events during the late stage scenario (Fig 8).

With planting improved cultivars early, the average yield was significantly increased to 7.09–7.34 t/ha across the three precipitation frequency scenarios within RCP4.5 climate for conventional treatment and was significantly increased to 7.57–7.89 t/ha for no-till treatment (Fig 8a, 8c and 8e, p < 0.05). Average maize yield was significantly increased to 6.53–7.07 t/ha for conventional treatment and to 7.24–7.52 t/ha for no-till treatment, when the adaptation practices were implemented under the three scenarios within RCP6 climate (Fig 8b, 8d and 8f, p < 0.05). By comparison, the historical average maize yield was 5.58 t/ha and 6.15 t/ha for conventional and no-till treatments, respectively (Fig 8).

**Fig 9:**
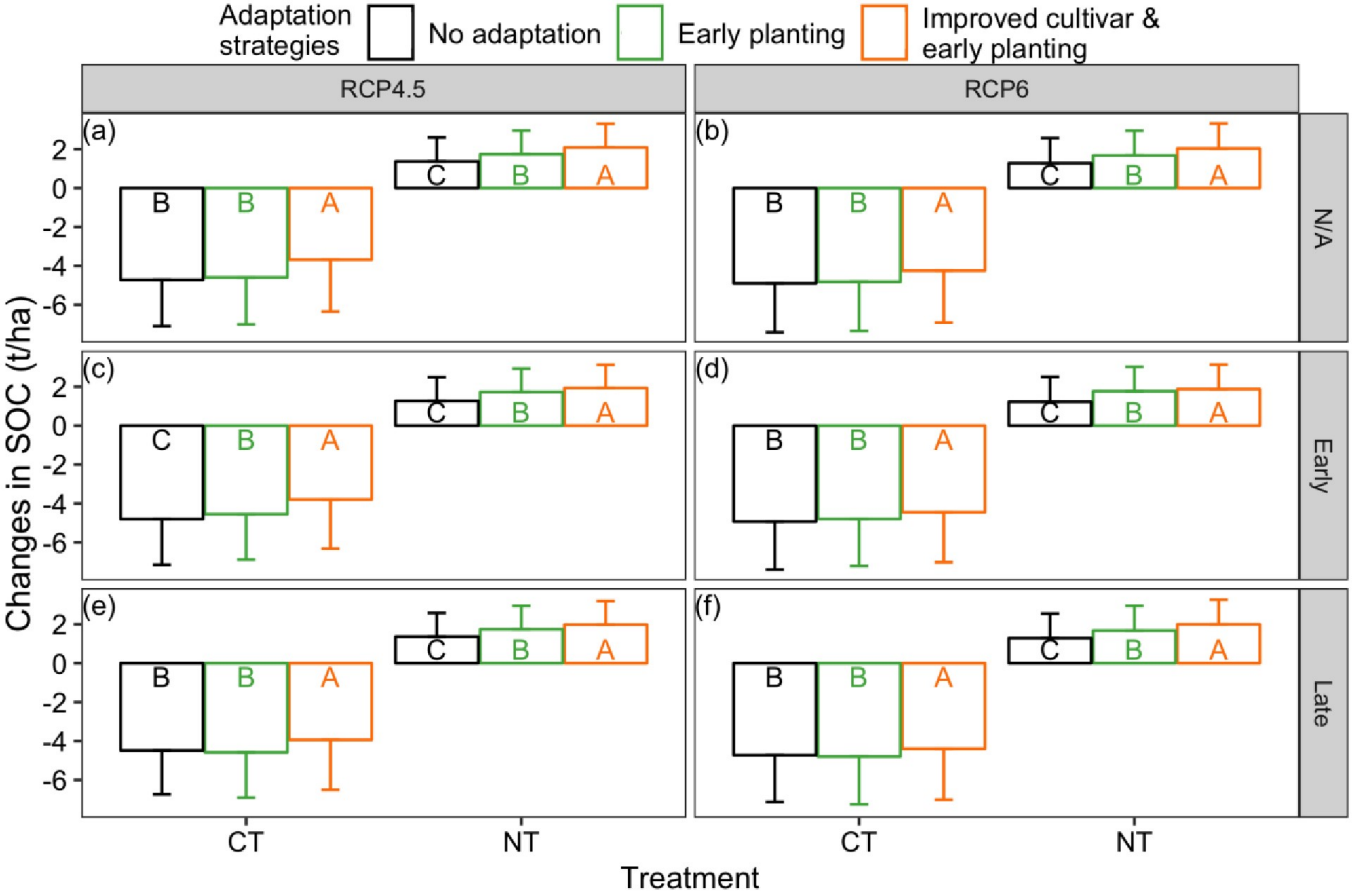
Average and standard deviation of the SOC with and without climate adaptation strategies under conventional tillage (CT) and no-till (NT) in the 28 years for various scenarios: (a-b) RCP4.5 and RCP6 climate without changing precipitation frequency, (c-d) RCP4.5 and RCP6 climates combined with changing precipitation frequency at maize and wheat early growth stage, (e-f) RCP4.5 and RCP6 climate combined with changing precipitation frequency at maize and wheat late growth stage (error bars represent the standard deviation; adaptation strategies sharing the same letter are not significantly different at P = 0.05 using Tukey’s honestly significant difference test within the tillage treatment).

Early planting adaptation practice reduced SOC loss under the conventional tillage to about 4.6 t/ha across the precipitation frequency scenarios under the RCP4.5 climate. Across the precipitation frequency scenarios under RCP6 climate, the adaptation strategy reduced SOC loss to about 4.8 t/ha. The smaller SOC loss brought by early planting was only significant under RCP 4.5 with extended drought followed by a storm event at early stage scenario (p < 0.05) (Fig 9). By comparison, without adaptation, the SOC loss from conventional treatments was between 4.48–4.81 t/ha and 4.73–4.93 t/ha across the scenarios within the RCP4.5 and RCP6 climates, respectively (Fig 9). SOC gain was slightly enhanced by the early planting practices for no-till treatment. SOC was significantly increased to 1.8 t/ha with the early planting adaptation practices across the RCP4.5 and RCP6 climates, compared to SOC gain of about 1.3 t/ha without adaptation practices for the no-till treatment (p < 0.05, Fig 9).

With the early planting of improved cultivar adaptation, SOC loss was decreased to about 4 t/ha under conventional treatment and SOC gain is increased to about 2 t/ha under no-till treatment across RCP4.5 and RCP6 climate scenarios combined with precipitation frequency scenarios (Fig 9). The enhanced SOC storage resulted from the two adaptation strategies were statistically significant when it compared to the no climate adaptation scenario (p < 0.05).

## 4. Discussion

Previous studies have discussed the mechanisms behind climate change impact on yield and SOC [9]. The increased CO_2_ concentration provides a fertilizer effect on both C3 crops (e.g. wheat) and C4 crops (e.g. maize), whereas the increased temperature and varied precipitation pattern lead to negative impact on crop growth and alter soil carbon dynamics. Extensive studies on grain production under climate change in the Midwest have shown decreased crop yield due to shortened growing season as a result of increased temperatures and SOC declines [5, 8, 9]. Our study also showed that the positive CO_2_ effect would not compensate the negative effect caused by the elevated temperature and less rainfall during growing seasons, as previously found by)[9]. Our results demonstrated that the effect of climate change on yield might be underestimated by overlooking at the effects of extreme precipitation events. The growth of summer food crops, such as maize, is sensitive to available soil water, which is dependent on the amount, timing, frequency and intensity of the rainfall. We included scenarios that counted the projected longer consecutive dry days [3, 48, 1], and we demonstrated that extreme precipitation events further decreased grain yield in the region in absence of adaptation strategies. Our findings are in agreement with previous research, which also showed that grain yield is more vulnerable to reduced rainfall at grain-fill stage than at vegetative stage [21, 49, 50]. Our study, nonetheless, had one inconsistent case where the extreme precipitation event caused more wheat yield reduction when it occurred in early season than in late season under RCP4.5 and RCP6 climates. This was due to forward shifts in wheat developmental stage under climate change. Wheat might be in reproductive stage under the projected climate when extreme precipitation events were imposed during the early-season, which were defined under historical climate.

No-till management is one of the pillars of conservation agriculture [51, 52]. No-till management imposes minimal disturbance to soil and previous crop residues retained on the soil surface slow down organic matter decomposition and increase water infiltration and decrease surface runoff [53, 54, 55]. Field experiments in different eco-zones across the globe have demonstrated that no-till management could lead to yield benefits, declines in yield or no change in yield, compared to conventional tillage management [56, 57]. A global metadata analysis concluded that the yield improvement led by no-till management was the most visible under rainfed condition in the dry climates [12]. We also found that no-till treatment produced a significantly higher average grain yield than conventional tillage treatment under the projected climate when available water becomes a limiting factor, but no-till management did not show significant enhancement on grain yield under historical climate in 1989–2016 (S2 and S5 Figs). Such benefit in grain yield was brought about by nutrient and water conservation under no-till management [58, 59]. Maize and wheat experienced less water deficiency stress under no-till than conventional tillage during the growing season in the three climate and precipitation scenarios (S8 and S9 Figs). More importantly, under climate change with more frequent prolonged drought and storm events, no-till management will play an important role in increased infiltration, reduced surface water loss by runoff and increased plant available water.

Soil carbon dynamics is interconnected with climate, tillage management and crop growth. This study confirms what was also found by)[9] that SOC would decrease more under climate change scenario compared to the historical climate for expedited soil carbon mineralization and reduced crop growth. Field experiments and simulation modeling studies also indicated the association between declined SOC and warming temperature [9, 60, 43, 17] showed that about 30% more carbon inputs were needed to maintain SOC in the temperate agricultural fields if temperature were to increase by 3.3 °C.

The need for management adaptation to climate change is certain, even though climate prediction is uncertain. The projected climate for the Midwest was a 2–8 °C increase in temperature and a 10–20% increase in total annual precipitation by the end of the 21^st^ century, depending on the greenhouse gas representative concentration pathways [3, 46]. Despite the uncertainties produced by climate models, elevated CO_2_, increased temperatures, longer drought periods, more heavy precipitation events are more likely to occur [61]. While others reported that planting maize earlier was effective in coping with the warming trend for the Midwest [62], we found that it is not sufficient to maintain maize yield under the projected RCP climates, unless adaptation strategies are implemented. Planting early would gain extra growing degree days but it may not be sufficient to compensate the loss in biomass accumulation due to higher temperature and faster development rate. We found that only planting the improved maize cultivar with longer duration and higher kernel numbers per ear could eliminate the adverse climate impact on maize grain production as shown in [23]. We also showed that with the improved maize cultivar and no-till management strategy, agricultural land could be a carbon sink and contribute to climate change mitigation. But the linkage between SOC and crop yield and crop residue biomass in the projected climate varies depending on climate zones, soil properties and the length of no-till practices [63, 43, 64].

## 5. Conclusions

Maize-soybean-wheat rotational cropping systems in the northern Midwest US are vulnerable to extreme weather events and climate change. Maize grain yield is projected to decline under the RCP4.5 and RCP6 climates, compared to the historical climate, whereas wheat grain yield will increase under the projected two climate scenarios. We found that additional SOC loss occurred under the two projected climate scenarios, compared to the historical climate, for both conventional tillage and no-till treatments. The extreme events (extended drought followed by heavy rainfall events) occurring at either early or late growing season will likely cause reduction in maize and wheat yield. The reduction in grain yield caused by extreme precipitation events was larger when the extreme precipitation events occurred in the late season than in the early season for maize.

No-till management systems cannot eliminate climate impact on the rainfed rotational cropping system. Adaptation strategies have to be adopted to mitigate climate change impact on cropping systems. We showed that planting early and adopting improved maize cultivars characterized by longer duration and more kernels, could mitigate the adverse impact of climate change on grain yield and SOC.

## Supporting information

S1 Fig Summary of consecutive dry days in the (a) wheat early growth stage, (b) maize early growth stage, (c) wheat late growth stage and (d) maize late growth stage in 1988–2017 (zero consecutive dry day was not displayed). (PDF)

S2 Fig Average maize yield under conventional tillage (CT) and under no till (NT) treatments in the simulated 24 years for various projected climates with various precipitation frequency scenarios: (a-c) historical, RCP4.5 and RCP6 climates without changing precipitation frequency, (d-f) historical, RCP4.5 and RCP6 climate combined with changing precipitation frequency at maize and wheat early growth stage, and (g-i) historical, RCP4.5 and RCP6 climate combined with changing precipitation frequency at maize and wheat late growth stage (the bar represents the standard error, n.s. denotes not significant at P = 0.05). (PDF)

S3 Fig Average maize yield under conventional tillage (CT) and under no till (NT) treatments in the simulated 24 years for various scenarios combining historical, RCP4.5 and RCP6 climates with various precipitation frequency scenarios: (a-c) historical, RCP4.5 and RCP6 climates under CT, (d-f) historical, RCP4.5 and RCP6 under NT (the bar represents the standard error; precipitation frequency scenarios, where drought and heavy rainfall events were not imposed (N/A) and imposed at early or late stage, sharing the same letter are not significantly different at P = 0.05 using Tukey’s honestly significant difference test). (PDF)

S4 Fig (a) Average maize yield under conventional (CT) treatment for the different climate scenarios considering three CO_2_ pathway climates, (b) the relative percentage change in the average maize yield under CT between climate scenarios with precipitation-frequency alteration at early or late growth stage and the respective climate without changing precipitation frequency, (c) average maize yield under no-till (NT) treatment for the different climate scenarios considering three CO_2_ pathway climates, (d) the relative percentage change in the average maize yield under NT between climate scenarios with precipitation-frequency alteration at early or late growth stage and the respective climate without changing precipitation frequency. (PDF)

S5 Fig Average wheat yield under conventional tillage (CT) and under no till (NT) treatments in the simulated 24 years for various projected climates with various precipitation frequency scenarios: (a-c) historical, RCP4.5 and RCP6 climates without changing precipitation frequency, (d-f) historical, RCP4.5 and RCP6 climate combined with changing precipitation frequency at maize and wheat early growth stage, and (g-i) historical, RCP4.5 and RCP6 climate combined with changing precipitation frequency at maize and wheat late growth stage (the bar represents the standard error, n.s. denotes not significant at P = 0.05). (PDF)

S6 Fig Average wheat yield under conventional tillage (CT) and under no till (NT) treatments in the simulated 24 years for various scenarios combining historical, RCP4.5 and RCP6 climates with various precipitation frequency scenarios: (a-c) historical, RCP4.5 and RCP6 climates under CT, (d-f) historical, RCP4.5 and RCP6 under NT (the bar represents the standard error; precipitation frequency scenarios, where drought and heavy rainfall events were not imposed (N/A) and imposed at early or late stage, sharing the same letter are not significantly different at P = 0.05 using Tukey’s honestly significant difference test). (PDF)

S7 Fig (a) Average wheat yield under conventional (CT) treatment for the different climate scenarios considering three CO_2_ pathway climates, (b) the relative percentage change in the average wheat yield under CT between climate scenarios with precipitation-frequency alteration at early or late growth stage and the respective climate without changing precipitation frequency, (c) average wheat yield under no-till (NT) treatment for the different climate scenarios considering three CO_2_ pathway climates, (d) the relative percentage change in the average wheat yield under NT between climate scenarios with precipitation-frequency alteration at early or late growth stage and the respective climate without changing precipitation frequency. (PDF)

S8 Fig Average maize and wheat cumulative early-stage water deficiency stress factor under conventional tillage (CT) and no till (NT) treatments in the simulated 24 years for various projected climates with various precipitation frequency scenarios: (a-c) historical, RCP4.5 and RCP6 climates without changing precipitation frequency, and (d-f) historical, RCP4.5 and RCP6 climate combined with changing precipitation frequency at early growth stage. (PDF)

S9 Fig Average maize and wheat cumulative late-stage water deficiency stress factor under conventional tillage (CT) and no till (NT) treatments in the simulated 24 years for various projected climates with various precipitation frequency scenarios: (a-c) historical, RCP4.5 and RCP6 climates without changing precipitation frequency, and (d-f) historical, RCP4.5 and RCP6 climate combined with changing precipitation frequency at late growth stage. (TIF)
