# Supplementary material for: Impacts of climate variability and adaptation strategies on crop yields and soil organic carbon in the US Midwest
Source: PLoS One. 2020 Jan 28;15(1):e0225433. doi: 10.1371/journal.pone.0225433 (PMC6986752; doi:10.1371/journal.pone.0225433)
Supplement: S1 Fig — (PDF) [file pone.0225433.s001.pdf]

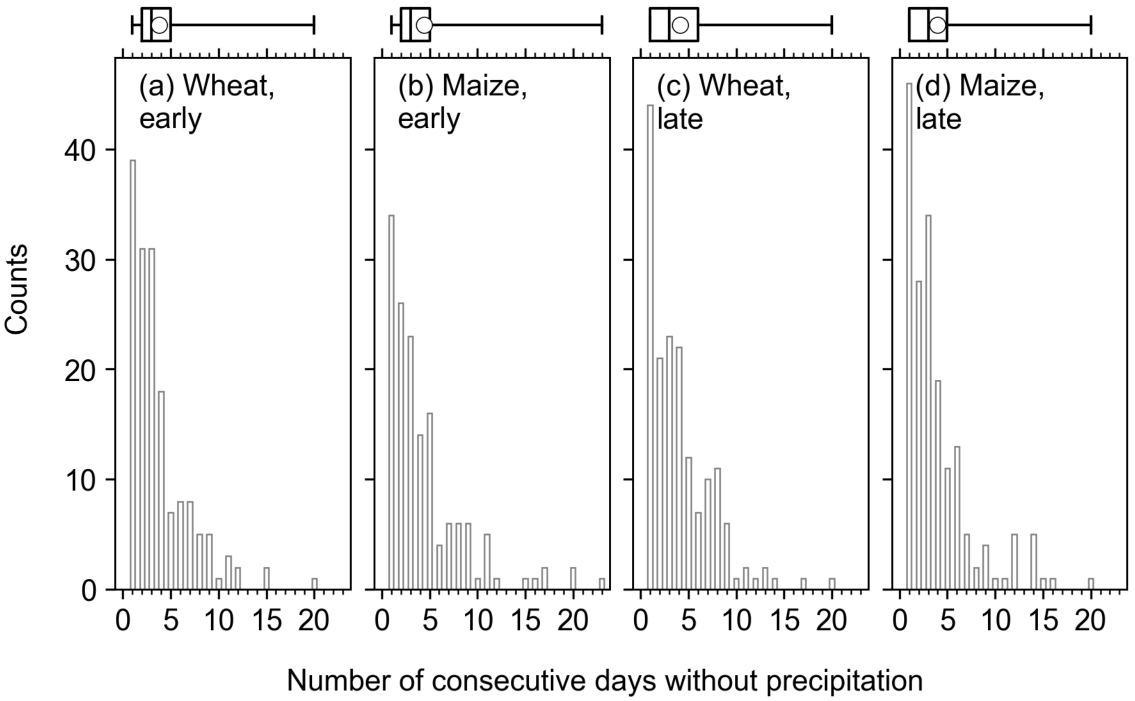

(a) Wheat, early
(b) Maize, early
(c) Wheat, late
(d) Maize, late
Counts
0
10
20
30
40
0 5 10 15 20
Number of consecutive days without precipitation
