# Supplementary figures and images for: Impacts of climate variability and adaptation strategies on crop yields and soil organic carbon in the US Midwest
Source: PLoS One. 2020 Jan 28;15(1):e0225433. doi: 10.1371/journal.pone.0225433 (PMC6986752; doi:10.1371/journal.pone.0225433)

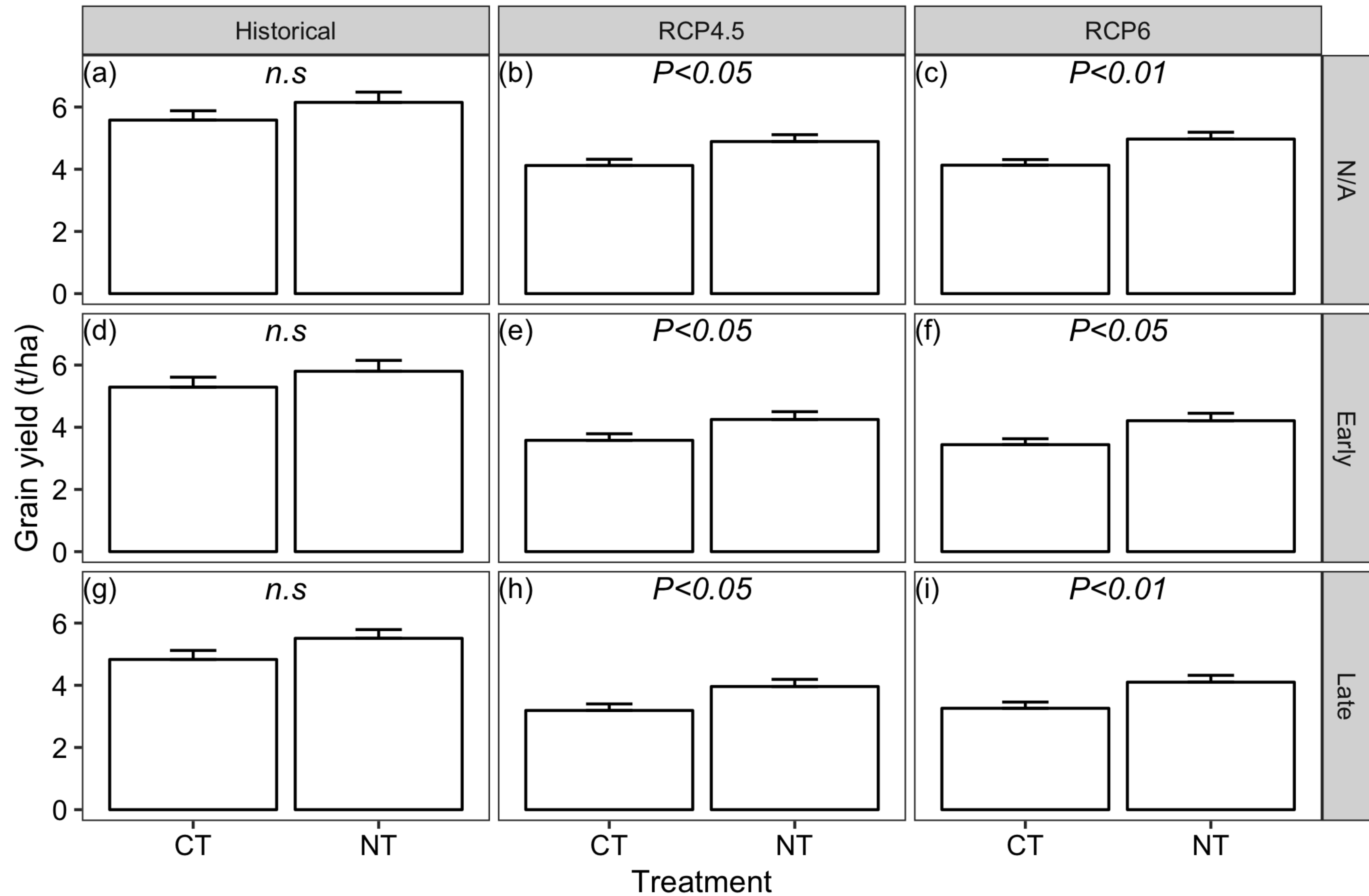

Supplement: S2 Fig — (PDF) [file pone.0225433.s002.pdf]

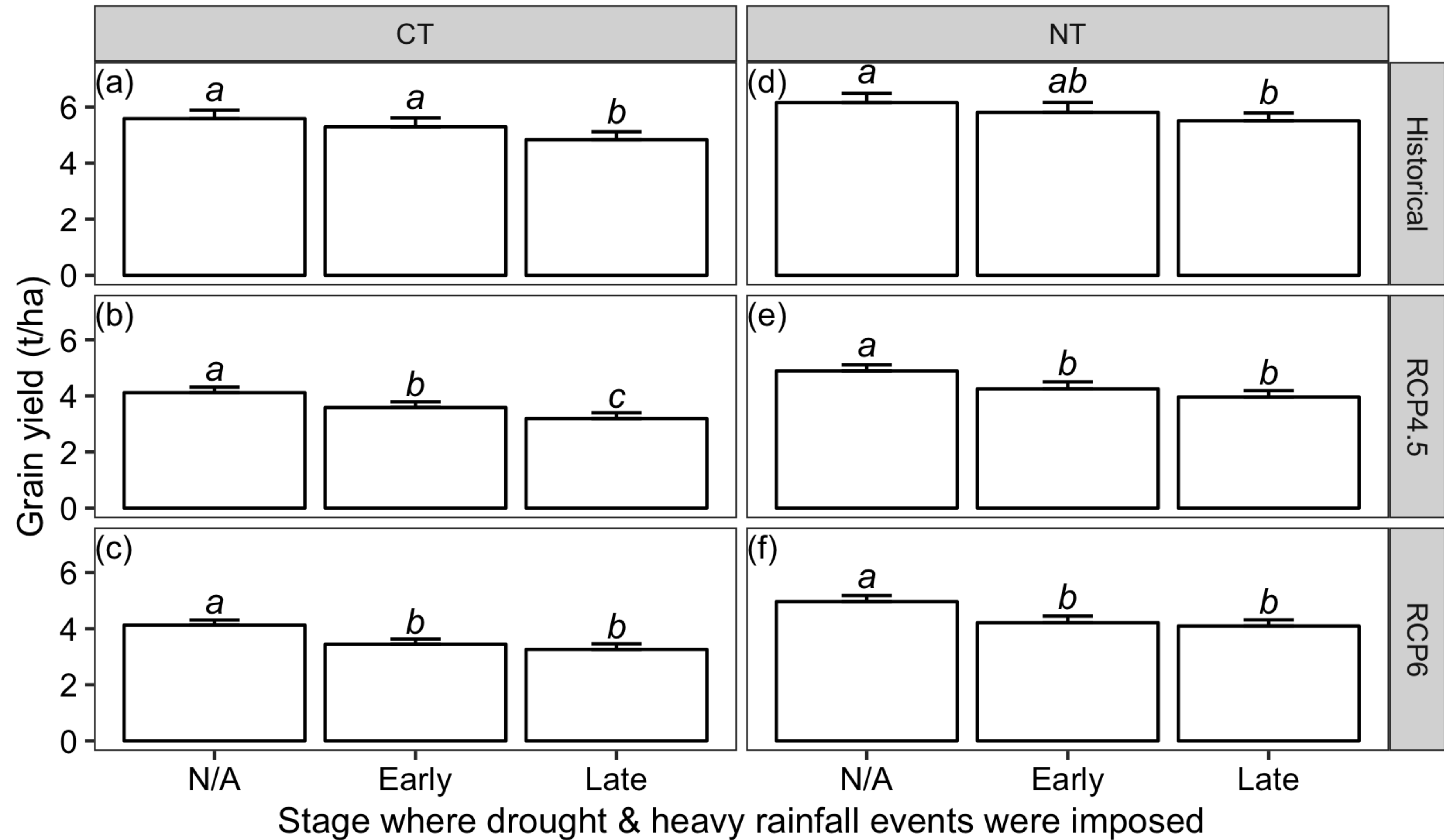

Supplement: S3 Fig — (PDF) [file pone.0225433.s003.pdf]

Stage where drought & heavy rainfall events were imposed

N/A Early Late

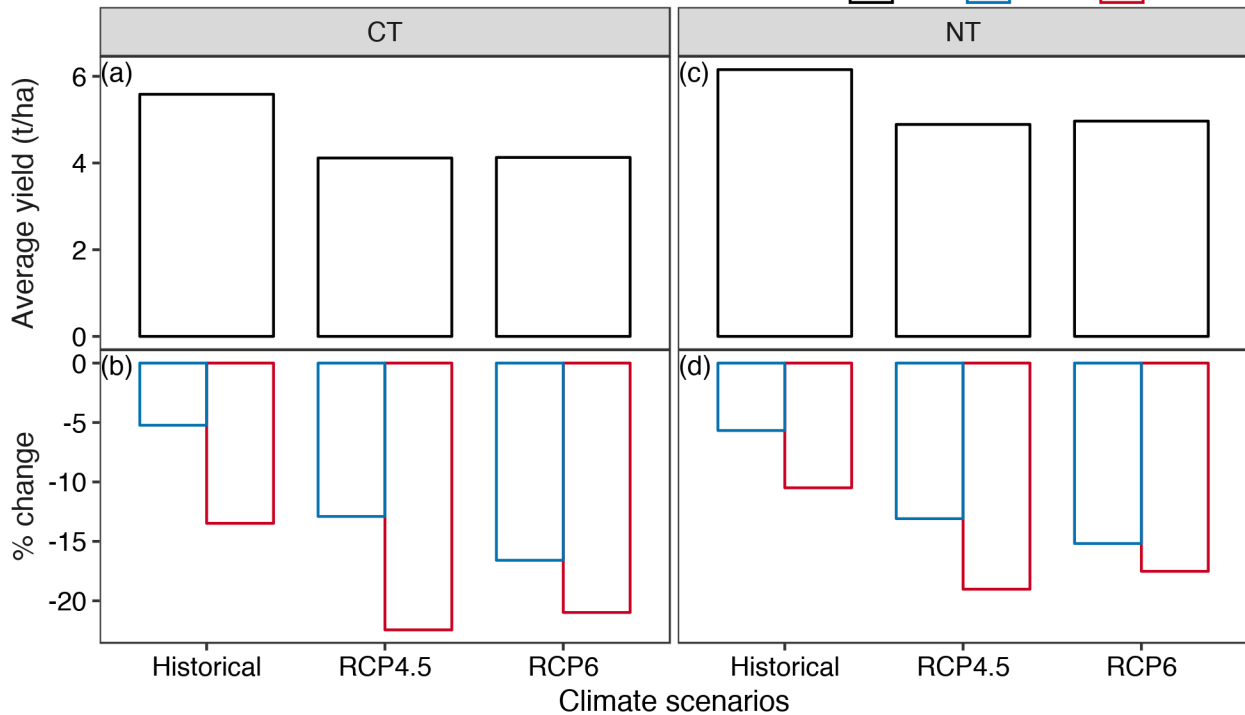

Supplement: S4 Fig — (PDF) [file pone.0225433.s004.pdf]

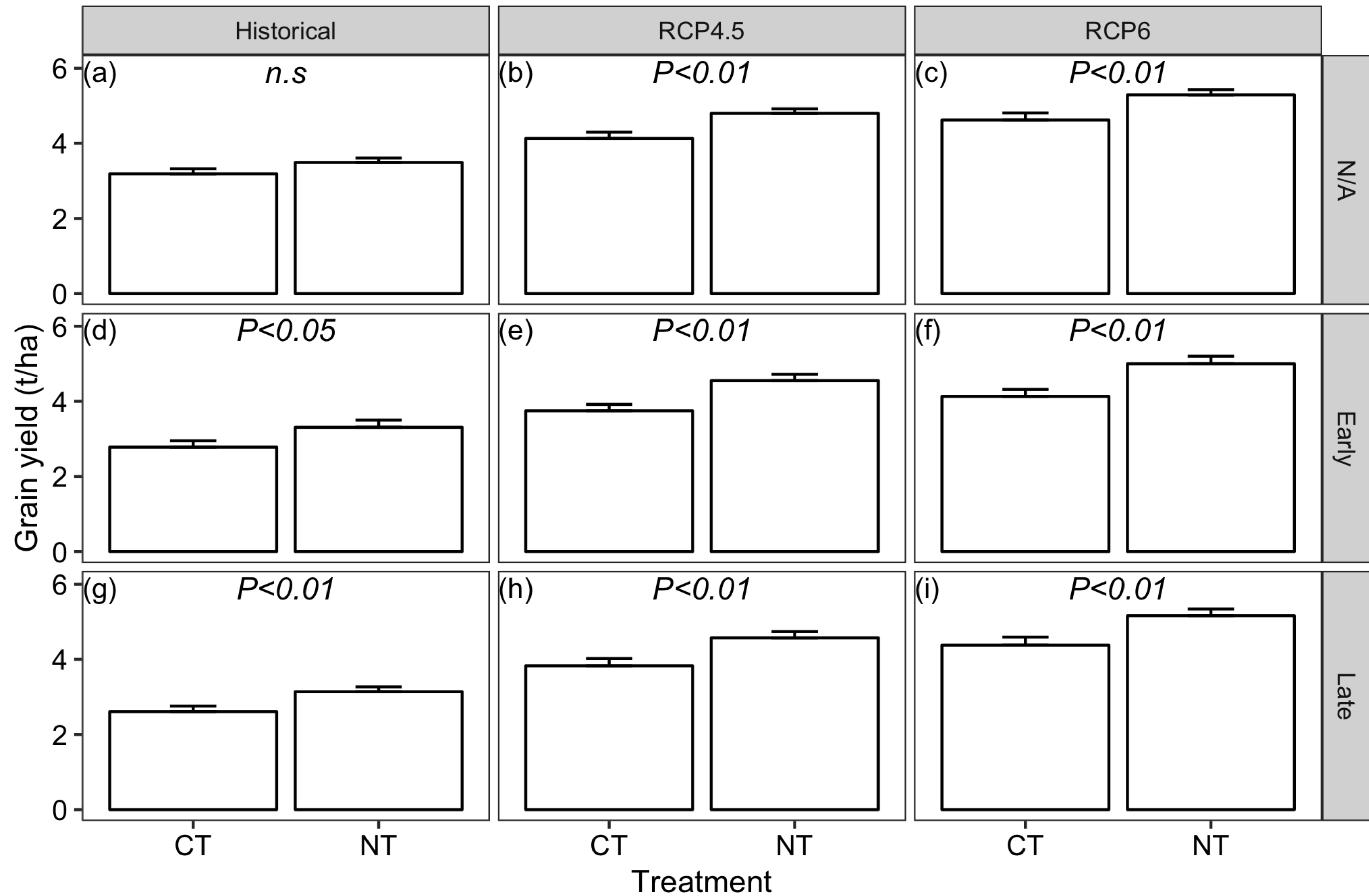

Supplement: S5 Fig — (PDF) [file pone.0225433.s005.pdf]

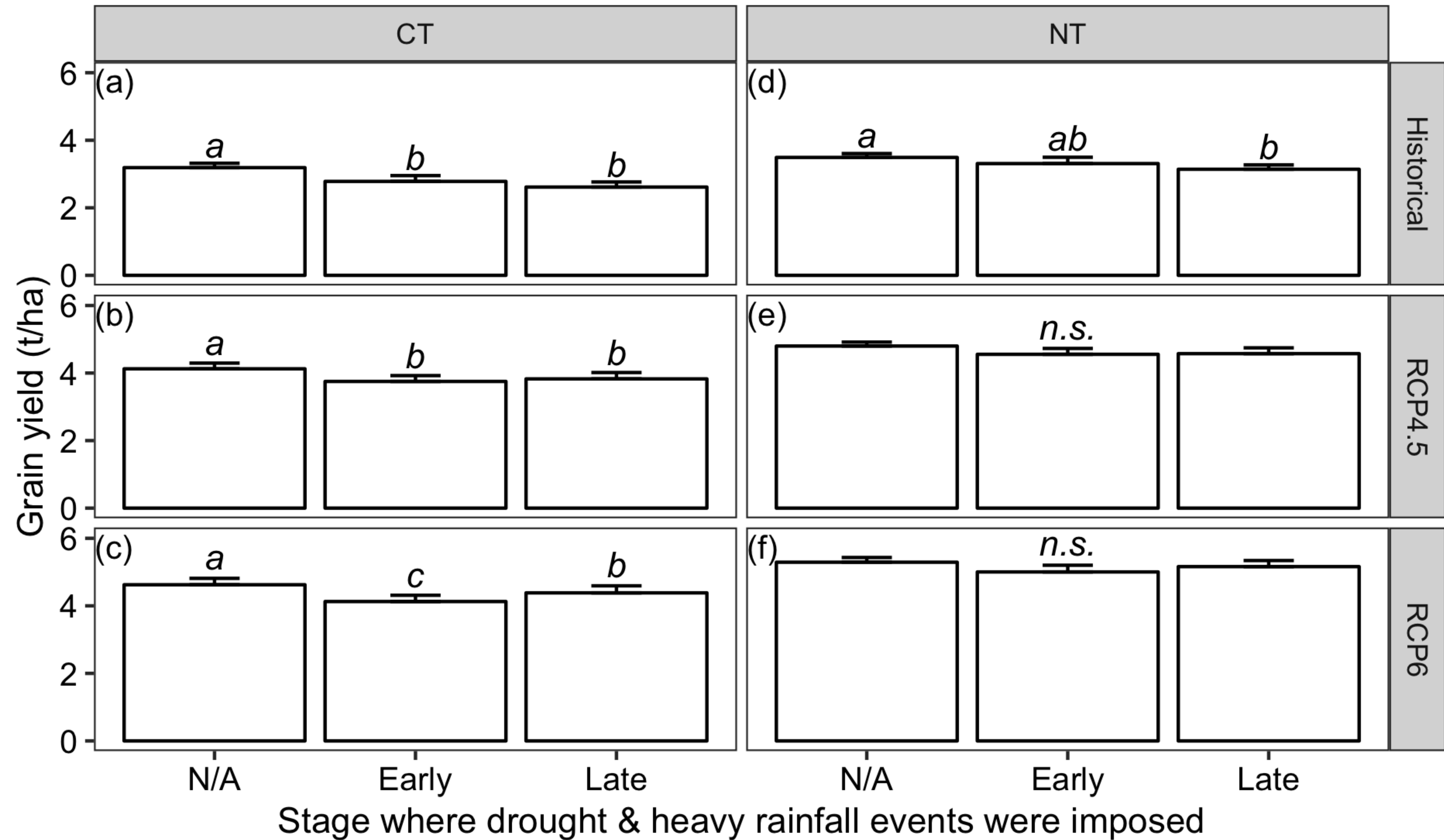

Supplement: S6 Fig — (PDF) [file pone.0225433.s006.pdf]

Stage where drought & heavy rainfall events were imposed  N/A  Early  Late

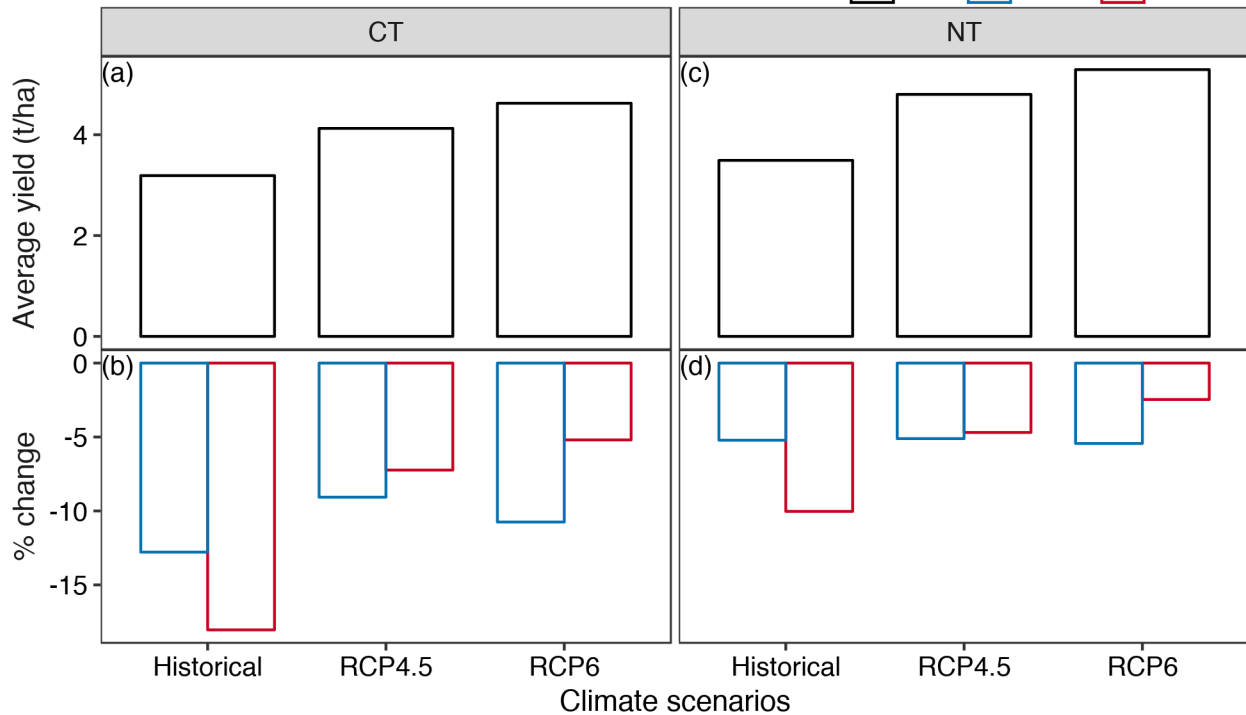

Supplement: S7 Fig — (PDF) [file pone.0225433.s007.pdf]

Treatment □ CT □ NT

Average cumulative water deficiency stress factor

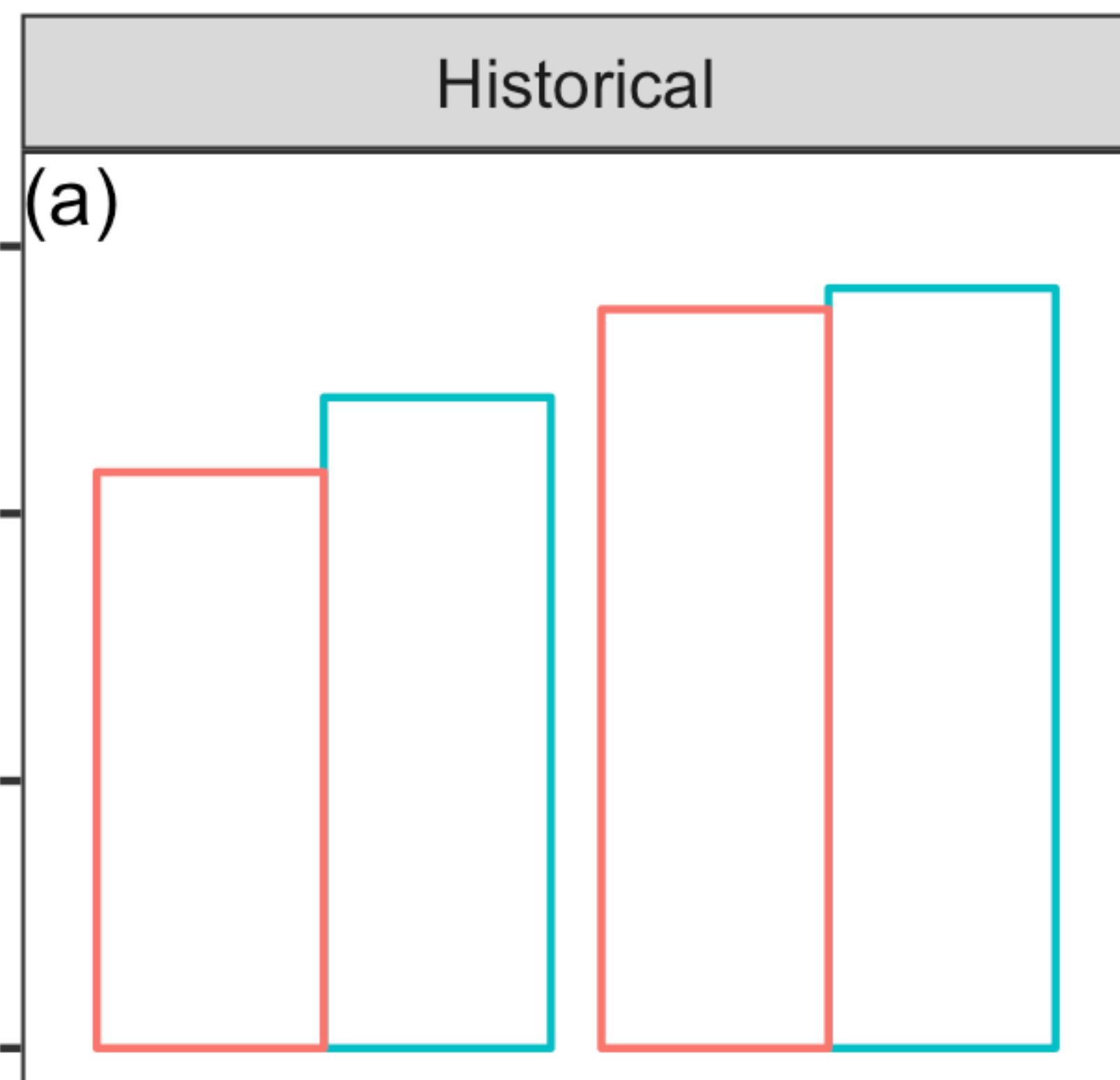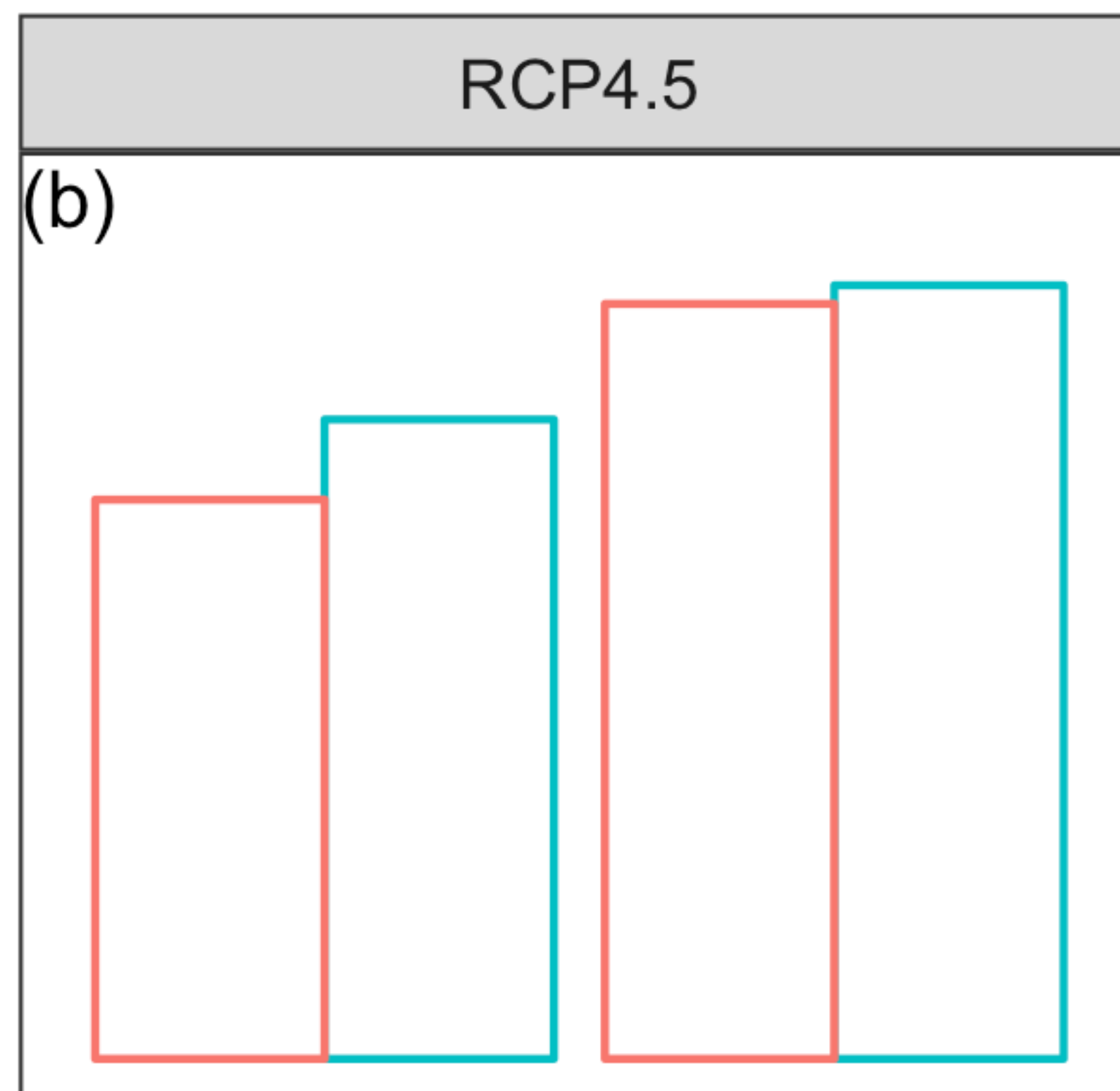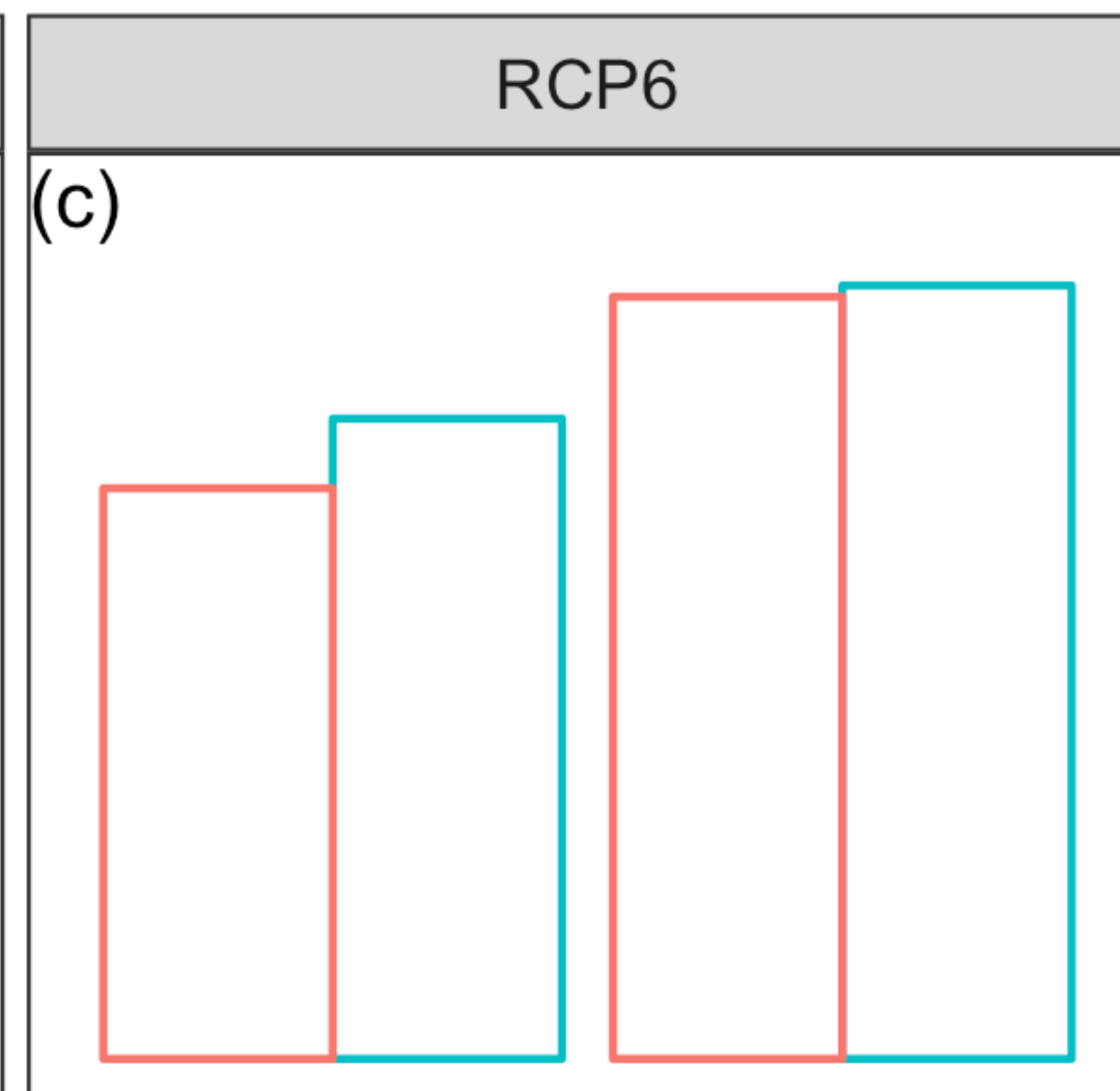

N/A

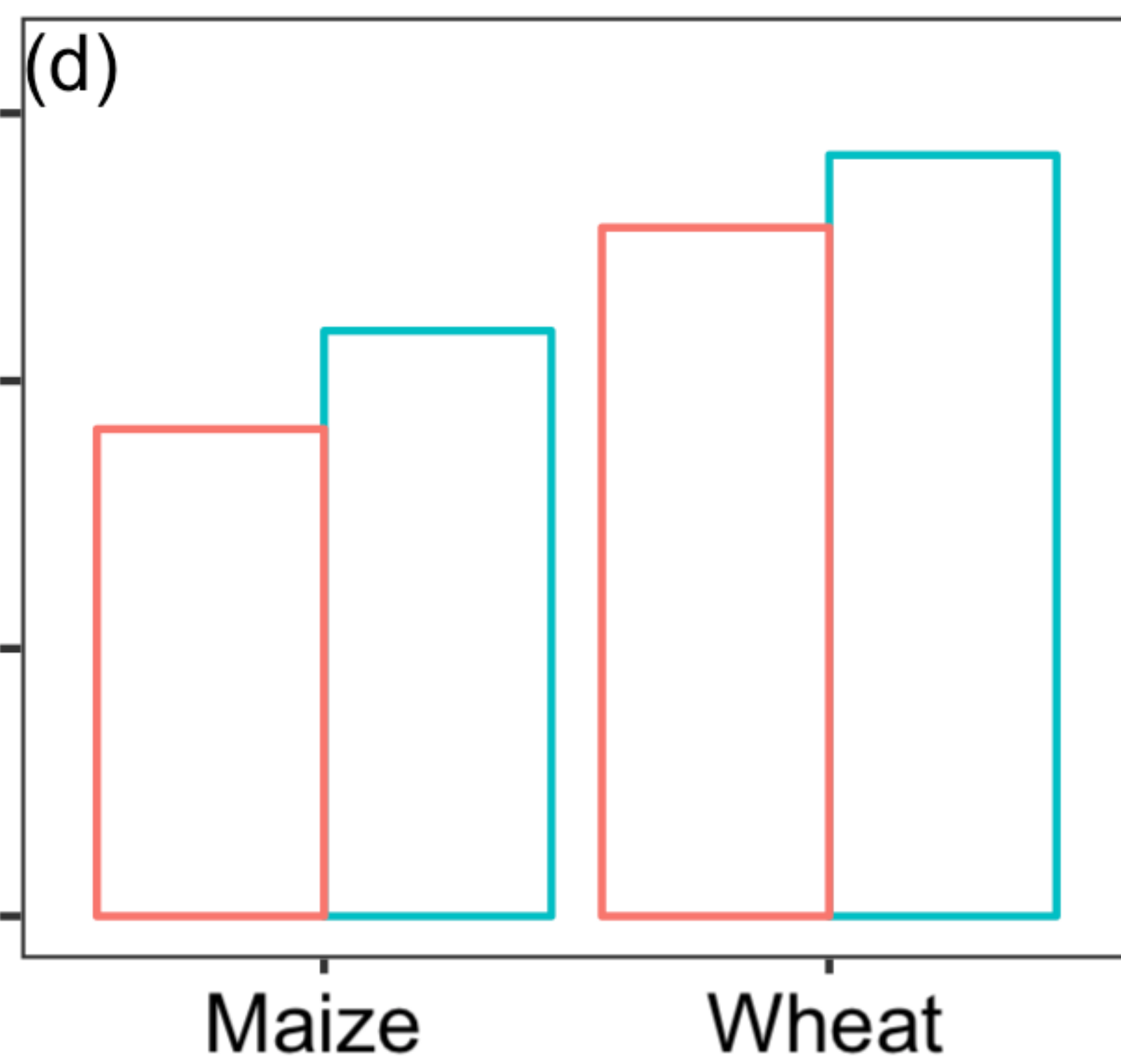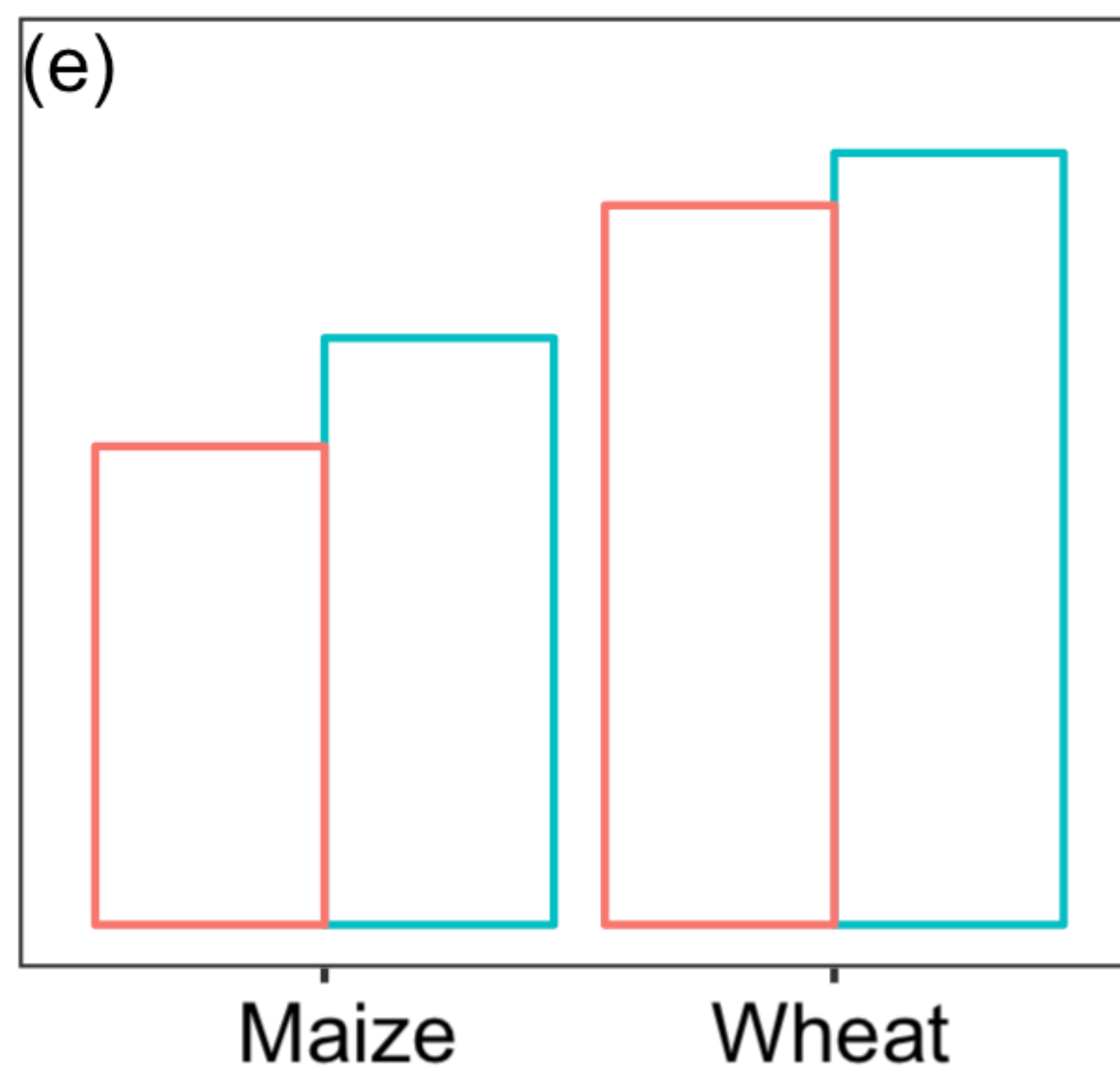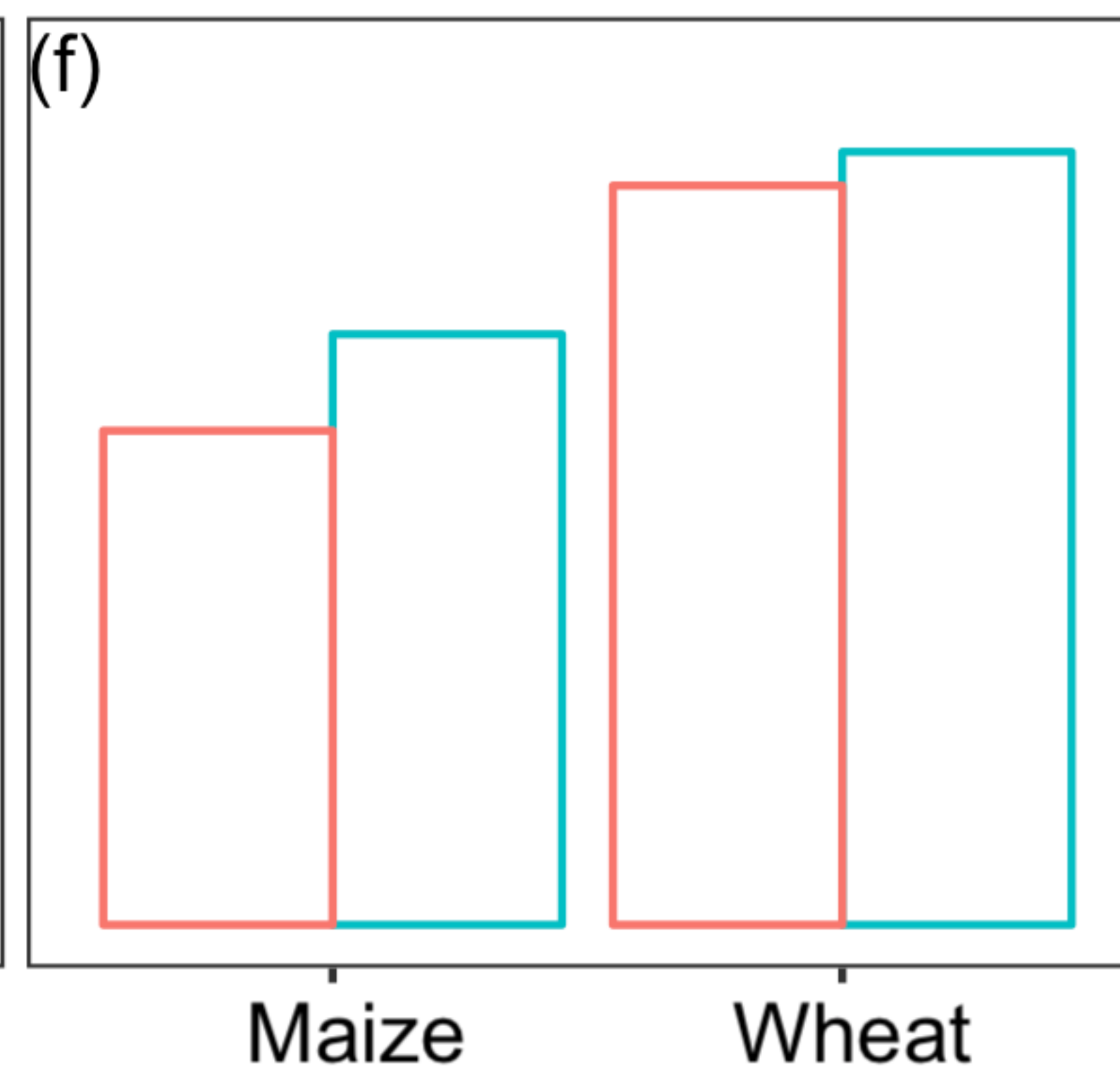

Early

Crop

Supplement: S8 Fig — (PDF) [file pone.0225433.s008.pdf]

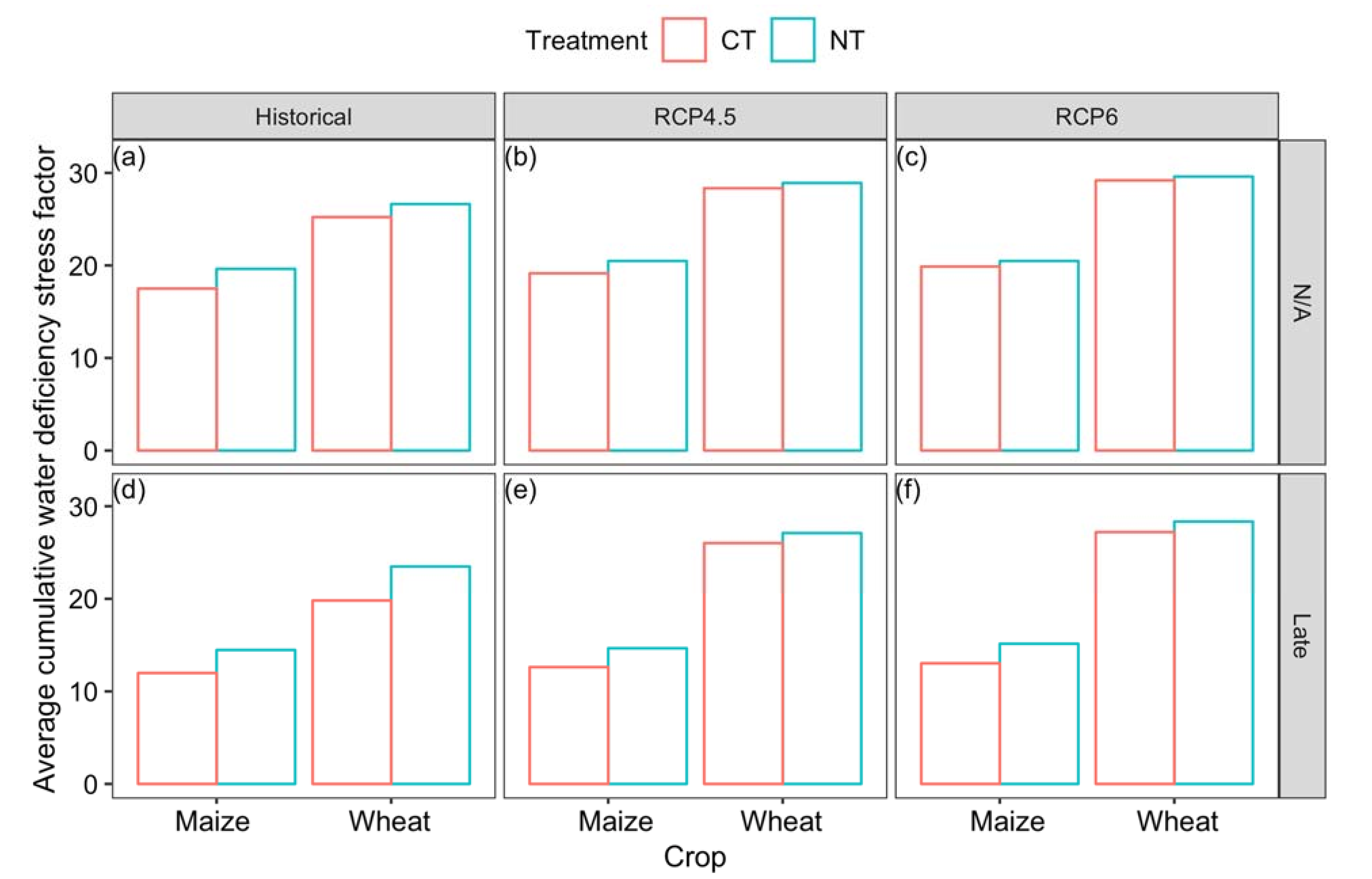

Supplement: S9 Fig — (TIF) [file pone.0225433.s009.tif]
